# 2D:4D and Self-Employment: A Preregistered Replication Study in a Large General Population Sample

**DOI:** 10.1177/1042258720985478

**Published:** 2021-01-05

**Authors:** Frank M. Fossen, Levent Neyse, Magnus Johannesson, Anna Dreber

**Affiliations:** 1 6851 Department of Economics, University of Nevada, Reno, USA; 2 Institute of Labor Economics, Bonn, Germany; 3 28354 SOEP at DIW, Berlin, Germany; 4 WZB, Berlin, Germany; 5 7648 Department of Economics, Stockholm School of Economics, Stockholm, Sweden; 6 Department of Economics, University of Innsbruck, Innsbruck, Austria

**Keywords:** self-employment, entrepreneurship, hormones, testosterone, digit ratio

## Abstract

The 2D: 4D digit ratio, the ratio of the length of the second finger to the length of the fourth finger, is often considered a proxy for testosterone exposure in utero. A recent study reported, among other things, an association between the left-hand 2D:4D and self-employment in a sample of 974 adults. In this preregistered study, we replicate the 2D:4D results on a sample of more than 2100 adults from the German Socioeconomic Panel-Innovation Sample (SOEP-IS). We find no statistically significant associations between 2D:4D and self-employment.

## Introduction

Understanding the determinants of self-employment (with and without employees) and entrepreneurship is important because entrepreneurs tend to innovate and prompt competition and thereby contribute to job creation and economic growth (e.g., Acs & Armington, 2006; Carree & Thurik, 2010; van Stel et al., 2005). Relatively recently, the potential roles of biological factors have received substantial interest in the entrepreneurship literature. For example, some studies have found that the tendency to engage in self-employment partly has a genetic basis (e.g., Lindquist et al., 2015; Nicolaou et al., 2018; Zhang et al., 2009). There is an even larger literature exploring the association between testosterone, a sex steroid, and self-employment. Some of these studies have explored circulating levels of testosterone with mixed results (e.g., Greene et al., 2014; Nicolaou et al., 2018; van der Loos et al., 2013; White et al., 2006).^1^ It has been argued that the combination of positive and null results could potentially be due to endogeneity between self-employment and testosterone since testosterone levels are not something fixed, but may react to, for instance, social context.^2^ Prenatal testosterone exposure in utero has thus been proposed to play a clearer causal role on self-employment through the impact of prenatal testosterone exposure on fetal brain development that in turn affects personality and preferences (Nicolaou et al., 2018). As testosterone exposure in utero is difficult to measure, various proxies have been proposed, with the most commonly used proxy being the ratio of the length of the second digit to the length of the fourth digit (2D:4D) on each hand (Manning et al., 1998). Supposedly, a lower 2D:4D digit ratio is an indication of higher testosterone exposure. As explored further in the Discussion section, the evidence of this supposed link between prenatal testosterone exposure and 2D:4D is mixed.

There have been many attempts to link 2D:4D to, for example, personality, various cognitive abilities and economic preferences including risk taking. The results are often contradictory (see e.g., Neyse, Vieider, et al., 2020; Parslow et al., 2019, for reviews), with mixed evidence for publication bias (e.g., Hilgard et al., 2019; Puts et al., 2008). There are also papers relating 2D:4D to economic outcomes outside the lab (e.g., Coates et al., 2009; Nye et al., 2017).

Only a few papers test for an association between 2D:4D and self-employment-related outcomes (Bönte et al., 2016; Nicolaou et al., 2018; Trahms et al., 2010; Unger et al., 2015). Bönte et al. (2016) study entrepreneurial intent in a sample of 432 German university students and find negative and statistically significant associations for the right-hand 2D:4D, and note that their “data indicate that the right-hand 2D:4D is more strongly related to both operationalizations of entrepreneurial intent” than left-hand 2D:4D. In a sample of 64 male German entrepreneurs, Unger et al. (2015) correlate 2D:4D and entrepreneurial impact as indicated by the number of employees and find no direct statistically significant association for either hand. However, they find for both hands a statistically significant interaction between 2D:4D and the psychological measure of need for achievement that predicts entrepreneurial impact. Trahms et al. (2010) study a sample of 90 American entrepreneurs and report statistically significant negative correlations between 2D:4D and strategic goal commitment and firm performance. It is, in this study, unclear whether they conducted the analysis using the average of both hands.^3^

The most relevant study to ours is Nicolaou et al. (2018)—this is the only previous study that examines the association between 2D:4D and actual self-employment. Nicolaou et al. (2018) use the largest sample prior to our study and test this relation for 450 men and 524 women separately (total *N* = 974) using survey data from Understanding Society’s Innovation Panel Wave 6 with data collected in 2013 on both self-employment and 2D:4D (this is part of the UK Household Longitudinal Study, a panel survey of individuals in the UK). Nicolaou et al. (2018) find that left-hand 2D:4D is statistically significantly negatively related to men’s self-employment (*p* < .01), whereas for women they report a “marginally significant” negative relation (*p* < .10). For the right-hand 2D:4D, they find no statistically significant associations and they do not report results for data pooled for men and women.^4^

The aim of this study is to as closely as possible replicate the analyses carried out in Nicolaou et al. (2018) on the association between 2D:4D and self-employment; but in a new and larger sample (*N* more than 2100; this is more than twice the sample size used in Nicolaou et al., 2018). Nicolaou et al. (2018) conducted two more studies that we do not attempt to replicate (which are not on 2D:4D). Our study was preregistered at OSF (https://osf.io/t94fv/), detailing all the analyses to be conducted before we had access to the complete dataset (we had access to the 2D:4D data before preregistration, but this dataset was merged with the data on self-employment after preregistration). The effect sizes in Nicolaou et al. (2018) for the left-hand digit ratio was a 5.8 percentage unit change in self-employment for men and a 3.3 percentage unit change in self-employment for women for a one standard deviation change in 2D:4D.^5^ Based on the estimated standard errors in our study, we have 80% power at the 0.5% statistical significance level to detect effect sizes of 56% (73%) the magnitude estimated by Nicolaou et al. (2018) for men (women).^6^

We find no statistically significant association between left-hand or right-hand 2D:4D and self-employment for either men or women. Our nonsignificant point estimates of a 1.56 percentage unit change in self-employment for men and a 0.03 percentage unit change in self-employment for women for a one standard deviation change in left-hand 2D:4D are more than 70% lower for men and more than 99% lower for women than those reported in Nicolaou et al. (2018). In addition to estimating results separately for men and women and separately for left-hand and right-hand 2D:4D as in Nicolaou et al. (2018), we also report preregistered results pooled for men and women as part of our primary analyses. By pooling data for men and women, we provide results for an approximately four times larger sample size than used in the separate analyses for men and women in Nicolaou et al. (2018). In the preregistered exploratory analyses, we also test if the association between 2D:4D and self-employment differs between men and women, but we find no statistically significant gender difference supporting that pooling the data is appropriate. In the pooled data, our estimated 99.5% confidence intervals are within a 2.5 percentage points change in self-employment for a one standard deviation change in 2D:4D. Larger effect sizes than this are thus unlikely. Smaller effect sizes than this may be considered economically important, and even larger studies are needed to rule those out. We perform several preregistered robustness tests related to, for instance, potential outliers. We find no statistically significant or even suggestive evidence for an association between 2D:4D and self-employment in these robustness tests.

The paper is organized as follows. We describe the study, including the sample and variables, and then we report the results. We end with a discussion.

## Data and Variables

Nicolaou et al. (2018) carried out logistic regressions with self-employment (1/0) as the dependent variable and 2D:4D as the independent variable of interest, and a number of control variables. Below, we describe these variables in our data and note any differences compared to Nicolaou et al. (2018).

### Sample and 2D:4D Digit Ratio Variable

The 2D:4D data were collected as part of another project (Neyse, Johannesson, et al., 2020) investigating the association between 2D:4D and economic preferences (risk taking, altruism, negative reciprocity, positive reciprocity, and trust). That study was preregistered prior to starting the data collection (https://osf.io/5vpdn/), including details about the data collection, measurement procedures, and statistical tests. Data on 2D:4D were collected between September 2018 and December 2018 in the German Socioeconomic Panel-Innovation Sample (SOEP-IS). SOEP is a longitudinal survey study that started in 1984 and that today has about 30,000 participants (Goebel et al., 2019). SOEP-IS, which we use, was established in 2012 and includes experimental and survey modules (Richter & Schupp, 2015). According to the 2018 release of SOEP-IS, it has a total number of 5722 participants from 3232 households, with 4860 individuals participating in the 2018 wave. The survey committee decided to get the 2D:4D data collected from 3958 participants, and since 2D:4D measurement was voluntary, a sample of 3482 participants with a right- or left-hand measure of 2D:4D was obtained (3433 participants with a right-hand measure, 3454 individuals with a left-hand 2D:4D measure, and 3405 individuals with an average measure of the right- and left-hand 2D:4D). Due to missing data on self-employment and control variables, we end up with a sample of *N* = 2151 for right-hand 2D:4D (*N* = 1021 for men and *N* = 1130 for women) and *N* = 2156 for left-hand 2D:4D (*N* = 1027 for men and *N* = 1129 for women). This is our analysis sample, where the sample size thus differs slightly depending on which hand is included.

Left- and right-hand 2D:4Ds of the participants were measured during the household surveys with the help of digital calipers. The initial reason for using calipers instead of flatbed scanners or mobile applications is the confidentiality of respondents. The SOEP survey committee explicitly warned us against collecting hand scans, which inevitably contain fingerprints of respondents. Furthermore, considering the fact that the interviewers would visit nearly 3000 households, calipers are more mobile- and time-efficient in comparison with scanners. To ensure the reliability of the measurements and minimize measurement errors, we first prepared a hand measurement protocol, which involved information on calibration of the calipers, preparations, seating positions, and the measurement process. The protocol was tested with research assistants and a coauthor of this study (Levent Neyse). Research assistants first measured a number of 2D:4D ratios using digital calipers. Then Levent Neyse measured the scans of the same hands separately using an image editing software (GIMP). The measurements were almost identical. Two hundred and sixty-three interviewers were trained for the 2D:4D measurements, with the hand measurement protocol posted on https://osf.io/5vpdn/.

While Nicolaou et al. (2018) excluded observations from the analysis if data were missing for the self-employment variable or any of the control variables, they did not exclude any 2D:4D measurements.^7^ We use the same approach in our main results, with the addition of two preregistered robustness tests with alternative definitions of our 2D:4D variable. Steps to prevent outliers due to mismeasurement or injured fingers were included in the interviewer instructions—interviewers were told that the typical 2D:4D range is between 0.8 and 1.1 and to repeat the measurement for values significantly outside this range. We thus have two recorded 2D:4D measurements for some individuals (available for right/left-hand 2D:4D for 90/149 individuals in the initial sample). In our main results, we included the first measurement, but we supplement this with a robustness test where the first measurement is replaced by the second measurement for the cases with two measurements. We refer to this robustness test as “the corrected sample.” Interviewers were also instructed to not measure hands for interviewees with missing or severely injured second digits (2D) or fourth digits (4D). However, based on interviewer comments in the data, it is clear that they sometimes still measured and commented on the injured hand. In our main results, we included all 2D:4D measurements even when there are such comments. We also supplemented this with a robustness test where outliers due to injured fingers were excluded—we excluded digit ratios outside the range of 0.8–1.2 (corresponding to +/−3–4 standard deviations away from the mean in our data). This excluded right/left-hand 2D:4D for 14/32 individuals in the original sample. We did this rather than trying to identify injured fingers from comments as the comments are not always clear (e.g., some comments mention crooked fingers without specifying whether these are caused by an injury). We refer to this robustness test as “the restricted sample.” By specifying the usual 2D:4D range to the interviewers and having a large number of robustness tests with corrected and restricted samples, we aimed to minimize the measurement errors that can be caused by physiological differences between respondents and by interviewers’ responses to those differences.

### Self-Employment Variable

The self-employment variable was generated from existing SOEP-IS data from 2018 (collected in the same year as the 2D:4D data). We followed a preanalysis plan posted prior to merging the datasets (https://osf.io/t94fv/)—the SOEP-IS data administration can confirm that the self-employment variables of the current study were not generated and linked to the 2D:4D data until the preanalysis plan had been posted.^8^

We defined a variable for self-employment that is as similar as possible to the one used by Nicolaou et al. (2018). The self-employment variable used by Nicolaou et al. (2018) was based on information about employment status in the year that 2D:4D (and the control variables included in the analysis) was measured as well as the employment status on the most recent job in previous years for those not working in the year of the data collection. See our preanalysis plan for more details on how the sample of Nicolaou et al. (2018) can be characterized. We do the same in our analysis: for those working in 2018, the self-employment question is based on the current job and for those not working in 2018 when our 2D:4D data were collected, we include information about the most recent job (if available).

In the SOEP data (including SOEP-IS), respondents are asked the following question (translated to English by the SOEP group): “What is your current occupational status? If you are employed in more than one position, please answer the following questions for your main position only.” We classify the response categories into the following three categories: “not working,” “self-employed,” and “working: not self-employed.”

We coded individuals that in the 2018 survey data were in any of the “self-employment” categories as 1 for “self-employed,” and we coded individuals that in the 2018 survey data were in any of the “working: not self-employed” categories as 0 for our self-employment variable. For those in the 2018 survey data who were in any of the response categories coded as “not working,” we checked if they were included in any previous SOEP data waves, and if they were, we used the same question as above to code them as “self-employed,” “working: not self-employed,” or “not working” in the previous waves and used information about self- employment for the most recent survey that they were coded as “self-employed” or “working: not self-employed.” If participants were coded as “not working” in the 2018 survey data and they were not included in any of the previous SOEP data collections, they were excluded from the analysis. If participants were coded as “not working” in the 2018 survey data and all previous SOEP data collections they were included in, they were also excluded from the analysis.

Note that we do not define “help in a family business” as being “self-employed,” as those who self-identify as helping family members are not usually considered self-employed. This response category was instead coded as “not working.” We also coded the response category “Military, Community Service” as “not working,” as this consists of individuals doing voluntary military or community service. We furthermore coded the following categories as “not working”: “Apprentice, Trainee Industry Technology,” “Apprentice, Trainee Trade and Commerce,” and “Trainee, Intern”—as an apprenticeship/trainee is somewhere in-between working and studying (and some of these individuals could potentially become self-employed). We included self-employed farmers among the group of self-employed in our primary analysis, but we also carry out a robustness test excluding self-employed farmers from the analysis (see below in the robustness tests section for more details).^9^ For further information on how we classify responses, see our preanalysis plan.

### Control Variables

When it comes to control variables, Nicolaou et al. (2018) included year of birth (as a continuous variable), education (as dummy variables with the following categories: no formal education, A-levels, college degree, other higher education), white (white = 1; nonwhite = 0), self-reported health (measured by a categorical variable from 1 = excellent to 5 = poor and included as a continuous variable between 1 and 5), urban (urban location = 1; nonurban location = 0), gross personal income (included as a continuous variable in euro 1000 per month), and handedness (1 = right handed; 2 = left-handed; 3 = ambidextrous; the variable seems to be included as a continuous variable between 1 and 3). Our control variables are similar with the following differences. We have neither a “white” variable nor a “handedness” variable in our data. In the Nicolaou et al. (2018) data, the correlations between these variables and self-employment and 2D:4D were low and the results are very similar when not controlling for those variables. Our dummy variables for educational attainment are (a) school education below Abitur (the German analog of A-levels) and no apprenticeship, (b) Abitur and/or apprenticeship (omitted base category), (c) college degree, and (d) vocational degree beyond apprenticeship. An urban area was defined as an urban settlement with a population of 10,000 or more in Nicolaou et al. (2018); in our case, it is defined as a city or district with 20,000 or more inhabitants. See our preanalysis plan for more details on our control variables. In Panel 1 of Table 1, we show descriptive statistics for self-employment, 2D:4D, and the control variables. We include a complete correlation matrix of these variables for the full sample in Panel 2 of this table; descriptive statistics and correlation matrices for men and women separately are provided in Online Appendix Tables S1 and S2 (the correlation matrices were not preregistered but suggested by the editor and one of the reviewers).

**Table 1:** Descriptive Statistics and Correlation Matrix for Men and Women Pooled.

	Self-employed	R2D:4D	L2D:4D	Mean 2D:4D	Female	Year of birth	Below Abitur	Abitur or apprenticeship	Other higher education	College	Health	Urban	Gross personal income
Panel 1: Descriptive statistics													
Mean	0.0946	1.0000	1.0017	1.0008	0.5237	1968.8	0.0779	0.4587	0.2110	0.2523	2.5093	0.5566	2.6344
SD	0.2928	0.0488	0.0681	0.0471	0.4996	14.576	0.2681	0.4984	0.4081	0.4344	0.8967	0.4969	2.2456
Min.	0	0.8002	0.3714	0.6709	0	1930	0	0	0	0	1	0	0
Max.	1	1.4474	2.2727	1.6513	1	2001	1	1	1	1	5	1	34
*N*	2156	2151	2156	2138	2156	2156	2156	2156	2156	2156	2156	2156	2156
Panel 2: Correlation matrix													
R2D:4D	−0.0218	1.0000											
	0.3138												
L2D:4D	−0.0330	0.2871	1.0000										
	0.1269	0.0000											
Mean 2D:4D	−0.0351	0.7240	0.8686	1.0000									
	0.1052	0.0000	0.0000										
Female	−0.0691	0.0337	0.0513	0.0543	1.0000								
	0.0014	0.1198	0.0177	0.0120									
Year of birth	−0.1641	0.0387	0.0354	0.0455	0.0267	1.0000							
	0.0000	0.0738	0.1016	0.0354	0.2180								
Below Abitur	−0.0642	0.0001	0.0126	0.0091	0.1008	0.0504	1.0000						
	0.0030	0.9965	0.5607	0.6736	0.0000	0.0197							
Abitur or apprenticeship	−0.0514	0.0066	−0.0180	−0.0095	−0.0672	−0.0152	−0.2669	1.0000					
	0.0175	0.7602	0.4055	0.6591	0.0019	0.4838	0.0000						
Other higher education	−0.0036	−0.0090	−0.0028	−0.0067	0.0725	0.0037	−0.1502	−0.4763	1.0000				
	0.8685	0.6761	0.8976	0.7575	0.0008	0.8632	0.0000	0.0000					
College	0.1018	0.0009	0.0155	0.0116	−0.0531	−0.0172	−0.1687	−0.5348	−0.3010	1.0000			
	0.0000	0.9682	0.4737	0.5916	0.0140	0.4266	0.0000	0.0000	0.0000				
Health	0.0274	0.0238	−0.0136	0.0025	−0.0032	−0.2514	0.0356	0.0637	0.0425	−0.1349	1.0000		
	0.2057	0.2721	0.5294	0.9084	0.8829	0.0000	0.0998	0.0032	0.0492	0.0000			
Urban	0.0094	−0.0026	−0.0170	−0.0136	0.0083	−0.0020	−0.0193	−0.0679	−0.0641	0.1499	0.0003	1.0000	
	0.6651	0.9036	0.4328	0.5303	0.7002	0.9275	0.3735	0.0017	0.0030	0.0000	0.9905		
Gross personal income	0.0995	−0.0294	−0.0122	−0.0240	−0.3132	0.0017	−0.1778	−0.1785	−0.0301	0.3425	−0.1426	0.0515	1.0000
	0.0000	0.1745	0.5731	0.2678	0.0000	0.9371	0.0000	0.0000	0.1638	0.0000	0.0000	0.0173	

*Note.* The descriptives for R2D:4D (L2D:4D) are for the *n* = 2151 (*n* = 2156) included in the primary analyses, and the descriptives for the mean 2D:4D are for the *n* = 2138 included in robustness test 1. The descriptives for self-employment and the control variables are for the *n* = 2156 included in the primary analyses for left-hand 2D:4D. Gross personal income is in euro 1000 per month. The correlation matrix shows correlation coefficients and *p* values of tests of significant differences from zero below. Here, we use all observations from our main estimations with right- and left-hand 2D:4D available (*N* = 2138).

## Results From Preregistered Analyses

All analyses reported in this section below were described in the preanalysis plan. We divided the tests in the preanalysis plan into primary hypothesis tests, robustness tests, and exploratory analyses. In line with recent recommendation of Benjamin et al. (2018), and as specified in our preanalysis plan, we refer to hypotheses tests with a *p* value below .005 as “statistically significant evidence” and tests with a *p* value below .05 as “suggestive evidence.” All tests report two-sided *p* values.

### Primary Hypotheses Tests

We report the results of the primary hypothesis tests in Table 2. For right-hand 2D:4D, Nicolaou et al. (2018) did not report any significant association between 2D:4D and self-employment for men or women. Consistent with this, we find no significant associations either. This is also the case when we pool men and women to increase statistical power further.

**Table 2:** Regression Analysis: Primary Hypotheses.

	Men	Women	Men	Women	Both	Both
R2D:4D	−1.879	0.338			−0.823	
	(2.231)	(2.305)			(1.591)	
L2D:4D			−2.934	−0.062		−1.084
			(1.949)	(1.400)		(1.171)
Female					−0.261	−0.237
					(0.161)	(0.160)
Year of birth	−0.049∗∗	−0.034∗∗	−0.049∗∗	−0.034∗∗	−0.043∗∗	−0.043∗∗
	(0.008)	(0.009)	(0.008)	(0.009)	(0.006)	(0.006)
Below Abitur	−0.976	−0.956	−0.964	−0.961	−0.871	−0.878
	(1.033)	(0.544)	(1.033)	(0.543)	(0.476)	(0.476)
Other higher education	0.340	−0.007	0.336	0.011	0.177	0.185
	(0.284)	(0.298)	(0.285)	(0.298)	(0.205)	(0.205)
College	0.743∗∗	0.452	0.756∗∗	0.501	0.594∗∗	0.614∗∗
	(0.246)	(0.295)	(0.246)	(0.292)	(0.186)	(0.185)
Health	−0.079	0.117	−0.068	0.119	0.022	0.028
	(0.126)	(0.125)	(0.125)	(0.125)	(0.089)	(0.088)
Urban	−0.147	0.100	−0.163	0.127	−0.038	−0.030
	(0.209)	(0.235)	(0.209)	(0.233)	(0.155)	(0.155)
Gross personal income	0.080∗	−0.037	0.082∗	−0.044	0.068∗	0.066∗
	(0.033)	(0.080)	(0.033)	(0.079)	(0.029)	(0.029)
Constant	96.729∗∗	63.480∗∗	96.284∗∗	64.061∗∗	82.289∗∗	82.056∗∗
	(16.414)	(17.534)	(16.337)	(17.397)	(11.936)	(11.879)
R2D:4D (ME)	−0.177	0.023			−0.067	
	(0.210)	(0.157)			(0.129)	
L2D:4D (ME)			−0.275	−0.004		−0.088
			(0.183)	(0.096)		(0.095)
Observations	1021	1130	1027	1129	2151	2156
Pseudo *R*^2^	0.099	0.041	0.101	0.042	0.074	0.074
χ^2^	72.44	24.55	73.67	25.70	99.64	100.24
*p*-value	0.000	0.002	0.000	0.001	0.000	0.000

*Note.* Logit regressions; standard errors in parentheses; *p < 0.05. **p < .005. (ME) shows marginal effects. Right and left 2D:4Ds are represented by R2D:4D and L2D:4D, respectively.

For left-hand 2D:4D, Nicolaou et al. (2018) reported a statistically significant (*p* < .01) association between 2D:4D and self-employment for men and a marginally significant (*p* < .10) association for women (using their terminology for statistical significance). The negative signs of these associations imply an effect in the direction of their hypothesis that higher prenatal testosterone is associated with a higher likelihood of being self-employed. In our replication, the point estimates are in the same direction, but the associations are not significant. Also, when we pool men and women the association between left-hand 2D:4D and self-employment is not significant. We thus fail to replicate their findings in the sense of finding a statistically significant (or suggestive) effect in the same direction.

It is also interesting to compare the estimated effect sizes between the studies. We compare the effect of a one standard deviation increase in 2D:4D on the probability of self-employment. Nicolaou et al. (2018) did not report marginal effects of their logistic regression coefficients, but we estimated the marginal effects based on their data to compare the effect sizes across the studies. We limit this comparison to left-hand 2D:4D where Nicolaou et al. (2018) reported significant associations. In Nicolaou et al. (2018), a one standard deviation increase in left-hand 2D:4D decreased self-employment by 5.8 percentage units for men and 3.3 percentage units for women. In our study, the corresponding effect sizes are 1.56 percentage units for men and 0.03 percentage units for women, and 0.6 percentage units if we pool men and women.^10^ Our nonsignificant point estimates of the effect sizes are thus more than 70% smaller for men and more than 99% smaller for women, compared to the effect sizes in Nicolaou et al. (2018).

**Figure 1:**
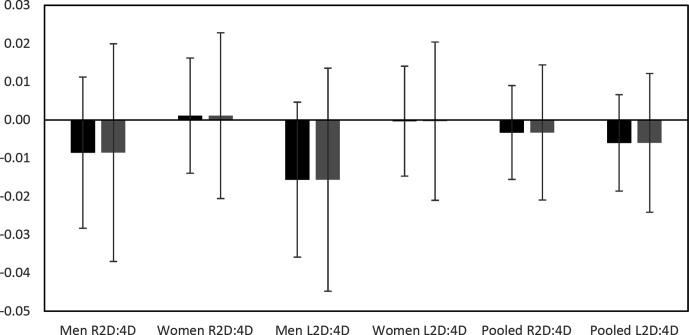
Effect sizes for primary hypotheses estimated by logit regressions. *Note*. 95% (left) and 99.5% (right) confidence intervals are presented. The units of the effect sizes are the percentage units changes in self-employment for a one standard deviation change in 2D:4D.

In Figure 1, we plot 99.5% and 95% confidence intervals for the effect sizes in the six regressions for our primary hypotheses tests (for a one standard deviation change in 2D:4D). The figure illustrates the precision of our estimates and shows what effect sizes we find strong evidence against (effect sizes outside the 99.5% confidence intervals). The upper bound of the 99.5% (95%) confidence interval is 4.5 (3.6) percentage units change in self-employment for men for left-hand 2D:4D.^11^ The corresponding upper bound for women is 2.1 (1.5) percentage units. For right-hand 2D:4D, the upper bound of the 99.5% (95%) confidence interval is 3.7 (2.8) percentage units change in self-employment for men and 2.1 (1.4) percentage units change for women, for a one standard deviation change in 2D:4D. When we pool men and women, the estimates get more precise and the upper bound of the 99.5% (95%) confidence interval is now 2.4 (1.9) percentage units change in self-employment for left-hand 2D:4D and 2.1 (1.6) percentage units change in self-employment for right-hand 2D:4D. Below, we also test the appropriateness of pooling men and women, by testing if there is a gender difference in the association.

### Robustness Tests

We carried out a number of preregistered robustness tests. In the first robustness test, we use the average of the left-hand and right-hand 2D:4D to increase the precision of the 2D:4D measurement. As shown in Table 3, there is no statistically significant or suggestive evidence of an association between 2D:4D and self-employment in these robustness tests, in line with the primary hypotheses tests.

**Table 3:** Robustness Test 1: Mean 2D:4D.

	Men	Women	Both
Mean 2D:4D	−3.663	−0.444	−1.842
	(2.525)	(2.203)	(1.673)
Female			−0.252
			(0.161)
Year of Birth	−0.049∗∗	−0.034∗∗	−0.043∗∗
	(0.008)	(0.009)	(0.006)
Below Abitur	−0.960	−0.954	−0.866
	(1.033)	(0.544)	(0.476)
Other higher education	0.336	−0.002	0.180
	(0.285)	(0.298)	(0.205)
College	0.741∗∗	0.453	0.592∗∗
	(0.246)	(0.295)	(0.186)
Health	−0.080	0.127	0.026
	(0.126)	(0.125)	(0.089)
Urban	−0.158	0.100	−0.042
	(0.209)	(0.234)	(0.155)
Gross personal income	0.081∗	−0.034	0.068∗
	(0.033)	(0.079)	(0.029)
Constant	97.819∗∗	64.258∗∗	83.056∗∗
	(16.384)	(17.496)	(11.921)
Mean 2D:4D (ME)	−0.346	−0.030	−0.150
	(0.239)	(0.151)	(0.136)
Observations	1015	1123	2138
Pseudo *R*^2^	0.101	0.041	0.075
χ^2^	73.78	24.88	100.89
*p*-value	0.000	0.002	0.000

*Note.* Logit regressions; standard errors in parentheses.

*p < .05. **p < .005. (ME) shows marginal effects.

In a second and a third robustness test, we estimate our results using the “corrected sample” and the “restricted sample” as detailed above (including also analyses on the average of the left-hand and right-hand 2D:4D), but this leads to similar results and does not affect our conclusions (the *p* value is >.05 in all these analyses). These results are reported in the Online Appendix Tables S3–6.

In a fourth robustness test, we estimate all our results using a linear probability model (estimated with robust standard errors) instead of logistic regressions. These results are estimated also for the “corrected sample” and the “restricted sample,” and for the average of the left-hand and right-hand 2D:4D. Like in the robustness tests 1–3, we find no statistically significant or suggestive evidence of an effect in any of these estimations pertaining to robustness test 4 either. All the results from robustness test 4 are reported in Online Appendix Tables S7–12. An advantage of the linear probability model is that it is easier to interpret the coefficients, and in Online Supplemental Figure S1 in the Appendix we show the 99.5% and 95% confidence intervals of the estimated effect sizes for the six regression models corresponding to the primary hypotheses tests in Table 2. These confidence intervals are similar to the ones shown in Figure 1.

In a final robustness test reported in Online Appendix Tables S13–24, we exclude self-employed farmers from the analyses, excluding nine observations in the analyses pooling men and women (five men and four women). This analysis without self-employed farmers is carried out for the primary hypothesis tests as well as for robustness tests 1–4. Again, we do not find statistically significant or suggestive evidence of an effect in any of these robustness tests.

### Exploratory Analyses

As a preregistered exploratory analysis, we tested if the association between 2D:4D and self-employment differs between men and women. We carried out these tests for the linear probability model as the coefficients are more straightforward to compare in that model, and we used a *z* test to test if the 2D:4D coefficient differed in the regressions for men and women. We did this test for the main sample included in Table 2 and for the analyses based on the average of the left-hand and right-hand 2D:4D, and for the “corrected sample” and “restricted sample” analyses. We also carried out this test for the robustness test excluding self-employed farmers. We find no statistically significant or suggestive evidence of a gender difference in any of these tests. This provides support for pooling the results of men and women as also done above. These test results are reported in Online Appendix Tables S25–26.

### Minimum Detectable Effect Size (Power)

As specified in the preanalysis plan, we also estimated the minimum detectable effect (MDE) sizes that we have 80% power to find at the 0.5% or 5% level. These estimations were based on the standard error of the 2D:4D coefficient in the linear probability models used in the robustness test, as the regression coefficients in the linear regression models are most straightforward to interpret (so that the units of the MDE is the percentage units change in self-employment for a one standard deviation change in 2D:4D). We estimated the MDE by multiplying the standard error of the 2D:4D coefficient by 3.65 (2.8) for 80% power to detect an effect at the *p* < .005 (*p* < .05) level. To further improve the interpretability of these results, we first multiplied the standard error of the 2D:4D coefficient by the standard deviation of 2D:4D for the sample included in the regression equation. We estimated the MDE for the primary hypothesis tests (the six regressions), but also for robustness tests 1–3 (the average of the left-hand and right-hand 2D:4D, the “corrected sample” and the “restricted sample”).

A useful benchmark for interpreting these results is the effect sizes reported in Nicolaou et al. (2018) where a one standard deviation change in left-hand 2D:4D increased self-employment by 5.8 percentage units for men and 3.3 percentage units for women. The MDE size estimations are shown in Online Appendix Table S27. In the different analyses, the MDE varies between 1.9 and 3.5 percentage units for tests at the 0.5% level and between 1.5 and 2.7 percentage units for tests at the 5% level. The MDE of the primary hypothesis test for left-hand 2D:4D for tests at the 0.5% (5%) level is 3.2 (2.5) percentage units for men, 2.4 (1.9) percentage units for women, and 1.9 (1.5) percentage units when men and women are pooled. We are thus very well-powered to detect effect size of the magnitude observed by Nicolaou et al. (2018); for men, we have 80% power to detect 56% of the effect size observed by Nicolaou et al. (2018) and for women we have 80% power to detect 73% of the effect size observed by Nicolaou et al. (2018) for tests at the 0.5% level. When we pool results for men and women, the power increases further, and the same is true for tests at the suggestive (5%) significance level.

However, given that the mean self-employment rate in our sample is 9.5%, smaller effect sizes than the MDEs would still be sizable in relative terms and may be considered economically important; our study does not provide strong evidence against such smaller effect sizes.

## Not Preregistered Robustness Tests and Exploratory Analyses

In this section, we conduct various supplemental analyses that we had not preregistered.^12^ As in the preregistered main analysis, we require a *p* value below .005 for significant and below .05 for suggestive evidence.

### Correlations and Limited Controls

We start with presenting raw correlations between self-employment (the dependent variable in our main hypotheses tests) and 2D:4D as well as the other independent variables. Table 1 and Online Appendix Tables S1; S2 show that there is no statistically significant or suggestive evidence of a correlation between the 2D:4D ratio of any hand and self-employment, neither in the pooled sample nor in the separate samples of men and women.

If the effect of 2D:4D on self-employment is indirect and transmits via one of our control variables, this potential indirect effect of 2D:4D may be hidden in our main hypotheses tests due to the inclusion of these potential mechanisms as control variables. For example, if 2D:4D influences educational choice and education has an effect on self-employment choice later in life, 2D:4D would appear to have no effect when we control for education. However, the raw correlations show that 2D:4D is not significantly associated with self-employment in the absence of any control variables. To assess this possibility further, we run logistic regressions based on the main estimation sample pooling both genders and only controlling for gender. We control for gender since it is exogenous and correlated with 2D:4D and potentially also self-employment. The results in Online Appendix Table S28 reveal no statistically significant or suggestive evidence of an effect of 2D:4D.

### Effect Heterogeneity With Respect to Age

The effect of 2D:4D may be stronger at a younger age than at an older age, when individuals have been subject to more intervening nonbiological influences (Bönte et al., 2017). To explore this possibility, we interact 2D:4D with age.^13^ We estimate this model by OLS to facilitate interpretation of the coefficient of the interaction term. Online Appendix Table S29 shows that there are no statistically significant or suggestive interaction effects of 2D:4D.

### Excluding Groups From the Sample

The self-employed are very heterogeneous. In this supplemental analysis, we use a narrower definition of entrepreneurship. We exclude self-employed farmers from the sample (as we also do in robustness check 4 reported above), and in addition, here we also exclude liberal professionals (self-employed physicians, lawyers, architects, journalists, translators, artists, and similar)^14^ and self-employed individuals not hiring any employees (own-account workers, independent contractors, and freelancers). Furthermore, hired managers might be considered similar to entrepreneurs, and including them in the comparison group might dilute the estimated effects. Therefore, we also exclude hired managers and executive civil servants in this set of estimations. Due to the narrow definition of entrepreneurship, the entrepreneurship rate is only 1.6% in the remaining sample. Online Appendix Table S30 presents the results. There is no significant or suggestive evidence of an association between 2D:4D and entrepreneurship.

### Ever Self-Employed and Entrepreneurial Intent

In our main hypotheses tests, the dependent variable indicates whether an individual’s current or last job was self-employment. In this subsection, we use two different dependent variables. First, we define a dummy variable that is one if an individual was ever self-employed within the observation period. The maximum period individuals in the SOEP-IS are observed is 1998–2018. Online Appendix Table S31 shows that there are no statistically significant or suggestive effects of 2D:4D on the probability of ever having been self-employed.^15^

Another outcome variable often used in entrepreneurship research is entrepreneurial intent. In every odd year between 1999 and 2011, the SOEP survey asked individuals to state the probability of becoming self-employed within the next 2 years, with choice options between 0% and 100% in intervals of 10%.^16^ We use this as a dependent variable measuring entrepreneurial intent, although in an imperfect way.^17^ For each individual with a measure of 2D:4D in the 2018 SOEP-IS, we use the newest observation of entrepreneurial intent when the individual was not self-employed and not older than 63 years. We estimate the model by OLS since the dependent variable is not binary. For each individual, we take the control variables from the same survey year when the dependent variable is observed. We control for age instead of year of birth to account for the fact that individuals report their entrepreneurial intent in different years. We use real gross personal income in prices of 2018 based on the Consumer Price Index (Federal Statistical Office of Germany, 2020).^18^ The estimation sample is substantially smaller than in our main hypotheses tests because of sample attrition between the years when the question on entrepreneurial intent was included in the questionnaire (1999, 2011) and the 2D:4D measurement in 2018. Online Appendix Table S32 shows that there are no statistically significant or suggestive effects of 2D:4D on entrepreneurial intent.^19^ The bivariate correlations of 2D:4D with entrepreneurial intent are not different from zero either in a significant or suggestive sense.^20^ Bönte et al. (2016) analyze the association between 2D:4D and entrepreneurial intent using a sample of students and argue that “entrepreneurial intentions of younger people with less professional experience and less commitment to specific occupations are less likely to be influenced by external factors not related to biology” (Bönte et al., 2016, p. 1128). To test if the effect is larger for younger individuals, we run additional regressions including interaction terms between 2D:4D and age (with the control variables). There is no statistically significant or suggestive evidence of an interaction between 2D:4D and age in these additional regressions.^21^

### Meta-Analysis

Lastly, we conduct fixed-effects meta-analyses (Borenstein et al., 2009) to combine the standardized marginal effects estimated in the primary hypotheses tests in this paper with those derived from Nicolaou et al. (2018). A meta-analysis implies evaluating the cumulative evidence for an hypothesis as recommended by, for instance, Schmidt (1996). We conduct meta-analyses for each hand for men, women, and both genders combined. Nicolaou et al. (2018) do not pool genders, so we first estimate pooled effects using their data based on logistic regressions analogous to those reported by these authors for men and women separately, and then we combine these pooled results with our pooled estimates using a meta-analysis.^22^ For each hand, we also test whether the estimated effects are significantly different between the two studies using a *z* test. The results appear in Online Appendix Table S33.

Note, first when we pool results for men and women in Nicolaou et al. (2018), the association between left-hand 2D:4D and self-employment is statistically significant (*z* = 3.196; *p* = .0014), but the association between right-hand 2D:4D and self-employment is not. For right-hand 2D:4D both studies thus reach the same conclusion of no evidence of an association between right-hand 2D:4D and self-employment and consistent with this the *z*-test of a difference in effect sizes between the 2 studies shows no statistically significant or suggestive evidence of a difference in effect sizes between the 2 studies. For left-hand 2D:4D we find suggestive evidence of a lower effect size in our study compared to Nicolaou et al. (2018) when the results of men and women are pooled, but we do not find statistically significant or suggestive evidence of a lower effect size in our study when men and women are analyzed separately. However, testing for a significantly lower effect size in the two latter comparisons is not a very informative replication criteria in our study. If the null hypothesis is true even replication studies that are much larger than the original study will be underpowered to find a significantly lower effect size than in the original study if the *p*-value of the original study is close to the threshold used for statistical significance (as the standard error of the difference in effect sizes between the replication study and the original study in the *z*-test will always exceed the standard error of the effect size of the original study). We illustrate this issue by also reporting the MDE size difference between the two studies that we have 80% power to detect at the 0.5% and 5% levels. As seen in Online Appendix Table S33, the MDE for suggestive evidence for left-hand 2D:4D for men and left-hand 2D:4D for women exceed the original effect size in Nicolaou et al. (2018), implying that we are underpowered to find suggestive evidence of a difference in effect sizes in these tests (and we are even more underpowered for finding statistically significant evidence of a difference). When results are pooled for the left hand of men and women, the MDE for suggestive evidence is similar to the original effect size in Nicolaou et al. (2018), showing that we are powered to find suggestive evidence of a difference in that test (but also in this test we are underpowered to find statistically significant evidence of a difference). Consistent with this, we find suggestive evidence of a lower effect size in our replication study in that test.

In the meta-analysis, we find no statistically significant or suggestive evidence of an association between right-hand 2D:4D and self-employment for men, women, or men and women pooled. For left-hand 2D:4D, we find no statistically significant association between 2D:4D and self-employment in any of the meta-analyses either, but we find suggestive evidence of an association for men and for men and women pooled (but not for women). The point estimate of the meta-analytic effect size for men is a 2.4 percentage unit decrease in self-employment for a one standard deviation increase in left-hand 2D:4D, and the corresponding point estimate for men and women pooled is a 1.3 percentage unit reduction in self-employment.

In interpreting the meta-analytic effect sizes, one should keep in mind that the original study by Nicolaou et al. (2018) was not preregistered. There is substantial evidence of inflated effect sizes in original studies that have not been preregistered. Several recent large-scale replication projects have been conducted in the social sciences, which found evidence of strongly inflated effect sizes on average in the original studies subject to replication (Camerer et al., 2016, 2018; Open Science Collaboration, 2015). On average, the original effect sizes were about twice as large as the replication effect sizes in these studies. These large-scale replication studies also reported fixed-effects meta analyses pooling the original effect size and the replication effect size. However, they also emphasized the limitations of such a meta-analysis, as effect sizes of original studies are typically inflated due to publication bias and selective reporting of results leading to inflated meta-analytic effect sizes. See also the recent study by Kvarven et al. (2020) for additional evidence of inflated effect sizes in meta analyzes. Our meta-analytic effect sizes should thus be interpreted very cautiously.

## Discussion

Studying the determinants of self-employment and entrepreneurship is important for better understanding who becomes an entrepreneur. A growing number of studies has investigated the importance of biological factors such as hormones and 2D:4D for self-employment and entrepreneurship. Here, we find no substantive evidence of an association between 2D:4D and self-employment, contradicting the conclusions for left-hand 2D:4D in Nicolaou et al. (2018). Our failure to find substantive evidence for the hypothesis that 2D:4D is associated with self-employment could be due to many reasons.

First, it is not clear that 2D:4D is actually a reliable proxy of testosterone exposure in utero. It has for example been argued that one piece of evidence comes from men having lower 2D:4D than women. While this often is the case, the gender difference is small with substantial overlap in distributions, and not all studies find a gender difference (Apicella et al., 2016). The most direct evidence is based on a sample of 29 children (boys and girls analyzed jointly) where there is a statistically significant negative correlation between the testosterone-to-estradiol ratio in amniotic fluid and right-hand 2D:4D, but not left-hand 2D:4D (Lutchmaya et al., 2004). In closely related studies, however, the results are less clear. For example, while Ventura et al. (2013) find a weak negative correlation between testosterone measured from amniotic fluid and 2D:4D of newborns for both hands among women (though only *p* < .10 for the right hand), there is no significant association among men in a sample of 51 women and 49 men. Ventura et al. (2013) do however find significant associations for both hands and both men and women using maternal plasma testosterone levels. There are more mixed and null examples from umbilical cord blood studies (Hickey et al., 2010; Whitehouse et al., 2015), with the largest study (*N* = 182 men and *N* = 159 women) finding no statistically significant associations (Hollier et al., 2015).^23^ There is also a theory of sex hormone transfer in utero, where female fetuses supposedly receive transfers of testosterone from their male co-twin (van Anders et al., 2006). Some studies thus compare the 2D:4D of same sex and opposite sex twins, where smaller studies report statistically significant associations while, again, larger studies do not (e.g., Hiraishi et al., 2012). Other indirect evidence includes the potential link between 2D:4D and Congenital Adrenal Hyperplasia (CAH, a disease that results in an excess production of testosterone) as well as CAG repeat polymorphism (which affects the transcriptional activity of the androgen receptor). While there are individual studies finding differences between individuals with and without CAH (e.g., Ökten et al., 2002), there are also many studies with null results (e.g., Nave et al., 2020), and a recent meta-analysis on CAH finds statistically significant correlations between right-hand but not left-hand 2D:4D and CAH in men and the opposite result for women (Richards et al., 2020). This meta-analysis also reports that compared to previous meta-analysis (Hönekopp & Watson, 2010), the average effect size was about half. The meta-analytic CAG results are instead null results (see e.g., Voracek et al., 2019). Men with Klinefelter’s Syndrome, a syndrome with testosterone deficiency as one of the main features, have however been shown to have higher 2D:4D (Manning et al., 2013) as “expected,” though the study is small with a sample of 51 men with Klinefelter’s Syndrome (these are compared to control groups). In sum, the evidence in support of a link between 2D:4D and prenatal testosterone exposure is not strong and it is not clear that 2D:4D is a valid proxy for testosterone exposure in utero. A potential explanation for our null results is thus that 2D:4D is not a valid proxy for testosterone in the utero. If that explanation is correct, prenatal testosterone exposure might play a role for self-employment but studies using 2D:4D cannot test that hypothesis.

Second, even if 2D:4D is a valid proxy of prenatal testosterone exposure, previous studies may have reported false positive results due to low power or small sample sizes, publication bias and “researcher degrees of freedom” (Gelman & Loken, 2013; Simmons et al., 2011). In most previous papers, the researchers test for statistically significant correlations in both hands but only find them for one hand—including Nicolaou et al. (2018) who only find significant results for the left hand and not the right hand—and look at both men and women but only find them in one group, and sometimes report results as “marginally significant” (*p* < .10). With this type of analysis and reporting, *p* < .05 results have high false positive probabilities. Moreover, if statistical power is low, there is even some chance that a statistically significant result is in the wrong direction from the true effect (Gelman & Carlin, 2014). Our sample size is more than twice the previously largest sample size on this topic (and when we pool men and women, there is a more than fourfold difference). With the hypothesis tests preregistered, there is also little room for researcher degrees of freedom affecting our results.

Third, it is also important to note that even though we find no statistically significant association between 2D:4D and self-employment, this does not imply that the null hypothesis is correct. Our estimated confidence intervals include potentially economically meaningful effect sizes in the direction found by Nicolaou et al. (2018), and an even larger sample size is needed to rule out such effect sizes. In pooling our results with the results of Nicolaou et al. (2018), we also find suggestive evidence of an association between left-hand 2D:4D and self-employment for men and for men and women pooled. In the meta-analyses including both men and women, the point estimates implies a 1.3 (0.3) percentage unit reduction in self-employment for a one standard deviation increase in left-hand (right-hand) 2D:4D. Our study is not well-powered to detect effect sizes of that magnitude, and such effect sizes can thus not be ruled out. Note also that the meta-analytic effect sizes should be interpreted very cautiously due to problems with inflated effect sizes in original studies resulting in overestimated effect sizes in the meta-analysis (Camerer et al., 2016, 2018; Kvarven et al., 2020; Open Science Collaboration, 2015).

Fourth, in order to replicate Nicolaou et al. (2018), we define the dependent variable, the control variables, and the sample as closely to this study as possible. Our failure to find significant effects does not rule out that 2D:4D is associated with other outcomes related to entrepreneurship such as entrepreneurial intent, as reported by Bönte et al. (2016) for university students; however, it is unclear in how far entrepreneurial intent among students will materialize in actual entrepreneurial behavior later in life. It is also possible that biological factors, including 2D:4D, affect younger persons more than older persons, who have been subject to more intervening nonbiological influences (Bönte et al., 2017). In an additional not preregistered exploratory analysis, we test for an association between 2D:4D and entrepreneurial intent in our data using a measure of entrepreneurial intent similar to one of the measures used by Bönte et al. (2016). We find no statistically significant or suggestive evidence of an association between 2D:4D and entrepreneurial intent in this analysis. However, the sample size used in this analysis is substantially smaller than in our primary hypotheses tests, and these null results are thus less informative. In further exploratory not preregistered tests, we also test for an interaction between age and 2D:4D, but find no evidence of such an interaction in our data. But it is clearly important to conduct further research on these issues.

Beside Nicolaou et al. (2018), the most relevant paper to ours is a paper by a subset of us (Neyse, Johannesson, et al., 2020). Using the same sample as us (but with *N* = 3482, which is larger since they do not match it to employment data), Neyse, Johannesson, et al. (2020) have previously explored to what extent 2D:4D correlates statistically significantly with risk taking, altruism, positive reciprocity, negative reciprocity and trust—all economic behaviors that had previously been related to 2D:4D albeit with mixed success and in substantially smaller samples with many researcher degrees of freedom. In a preregistered study, Neyse, Johannesson, et al. (2020) find no statistically significant association between 2D:4D and the five economic preferences.^24^

In sum, we fail to find substantive evidence for an association between 2D:4D and self-employment. The upper bound of the 99.5% confidence interval is a 2.4 (2.1) percentage point change in self-employment (pooling genders) for a one standard deviation change in left-hand (right-hand) 2D:4D. To rule out smaller effect sizes, even larger sample sizes are needed. Apart from large sample sizes, it is also crucial to preregister analyses plans in future studies to reduce researcher degrees of freedom.

## Supplemental Material

Table S1 - Supplemental material for 2D:4D and Self-Employment: A Preregistered Replication Study in a Large General Population Sample Supplemental material, Table S1, for 2D:4D and Self-Employment: A Preregistered Replication Study in a Large General Population Sample by Frank M. Fossen, Levent Neyse, Magnus Johannesson and Anna Dreber in Entrepreneurship Theory and Practice

## Notes

1. For example, White et al. (2006) report a positive association between circulating testosterone and new venture creation in a sample of 110 men, and Greene et al. (2014) find a positive association between testosterone and self-employment in a sample of 1119 men. In contrast, van der Loos et al. (2013) have two samples of 578 and 1697 individuals, respectively, and find no significant associations between testosterone and self-employment, while Nicolaou et al. (2018) find “marginal significance” (*p* < .0) for a positive association between predicted testosterone and self-employment among men (*N* = 1178) but not women (*N* = 968).

2. There is also work suggesting that the effects of testosterone administration on economic behaviors may depend on 2D:4D (e.g., van Honk et al., 2012).

3. Another indirectly related example is Guiso and Rustichini (2018) who look at a sample of 1313 Italian entrepreneurs and find that women in regions with less female emancipation on average have lower 2D:4D than men, while the opposite is the case in regions with higher female emancipation. These results are reported for both left- and right-hand 2D:4D. The authors interpret these results, as suggesting that there exist gender-related obstacles to entering entrepreneurship.

4. If anything, the literature suggests that right-hand 2D:4D is more commonly linked to both hormonal mechanisms and economically relevant behaviors (e.g., Hönekopp & Watson, 2010; Lutchmaya et al., 2004; Zheng & Cohn, 2011), but there are also examples of the opposite (e.g., Richards et al., 2020).

5. These effect sizes are based on our own estimations as Nicolaou et al. (2018) did not report marginal effects from their logistic regression analyses (we re-estimated their logistic regressions based on their posted data and added marginal effects to the estimation code).

6. Based on the recommendations of Benjamin et al. (2018), we preregistered 0.5% as the threshold for statistically significant evidence and 5% as the level for suggestive evidence.

7. Nicolaou et al. (2018) also carried out robustness tests based on winsorized results with 1% and 5% winsorization of the 2D:4D variable and reported these results to the reviewers during the review process, and the code for these analyses are also included in their posted code on Dataverse. But these results were not reported or referred to in the published paper. We thank the original authors of Nicolaou et al. (2018) for pointing out this. According to the original authors, their results were robust to 1% and 5% winsorization.

8. The questions can be addressed to SOEP-IS Survey manager, Prof. Dr. David Richter. E-mail: drichter@diw.de; Telephone: +49 30 89789 - 413; Address: Mohrenstraße 58, 10117 Berlin, Germany.

9. This robustness test excluding self-employed farmers was suggested by the original authors of Nicolaou et al. (2018) after reading a draft of our preregistration document.

10. These effects are obtained by multiplying the marginal effects in Table 2 by the standard deviations of 2D:4D in the corresponding estimation samples.

11. The upper bound here and below refers to the upper bound in absolute terms (as the point estimate of the 2D:4D coefficients is negative).

12. We thank the anonymous reviewers and the editor for suggesting these tests.

13. In the main hypotheses tests, we control for year of birth instead of age in order to closely follow Nicolaou et al. (2018). Here, we use age for ease of interpretation of the estimated coefficients when including the interaction term.

14. To be precise, we use a distinction defined in German law and well-known in Germany. In the SOEP, respondents are asked whether they are working as a liberal professional.

15. The bivariate correlations of 2D:4D with this variable are not different from zero either in a significant or suggestive sense. The correlation coefficients (*p* value) between right-hand 2D:4D and “ever self-employed” are –0.031 (*p* = .326) for men, 0.019 (*p* = .520) for women, and –0.009 (*p* = .686) for men and women pooled. The correlation coefficients between left-hand 2D:4D and entrepreneurial intent are –0.020 (*p* = .515) for men, –0.006 (*p* = .835) for women, and –0.009 (*p* = .669) for men and women pooled.

16. In 2011, the question was only posed to nonemployed individuals. The question was not included in the SOEP-IS in later years.

17. Bönte et al. (2016) use agreement with a similar statement (“I will start a business in the next five years”) as one of their measures of entrepreneurial intent. See also Thompson (2009) for the measurement of entrepreneurial intent.

18. Furthermore, gross personal income does not include alimony payments here because no consistent information is available for all years. In the main hypotheses tests, we included information on alimony payments available in 2018 to define the variable as closely as possible to the one used in Nicolaou et al. (2018).

19. The point estimates do not indicate economically important effect sizes either. The estimated effects of a change in 2D:4D by one standard deviation on the stated probability of becoming self-employed is small, as can be seen by multiplying the coefficients of 2D:4D by the standard deviations of 2D:4D, which are reported in Table 1.

20. The correlation coefficients (*p* value) between right-hand 2D:4D and entrepreneurial intent are –0.032 (*p* = .622) for men, 0.009 (*p* = .877) for women, and –0.013 (*p* = .772) for men and women pooled. The correlation coefficients between left-hand 2D:4D and entrepreneurial intent are –0.061 (*p* = .341) for men, –0.022 (*p* = .708) for women, and –0.048 (*p* = .259) for men and women pooled.

21. The OLS coefficients (standard errors) of these interaction terms are –0.031 (2.008) for male R2D:4D, 0.538 (1.631) for female R2D:4D, –0.389 (1.180) for male L2D:4D, 2.151 (1.315) for female L2D:4D, 0.507 (1.234) for pooled R2D:4D, and 0.511 (0.839) for pooled L2D:4D.

22. As for our primary hypotheses tests on men and women pooled we also include a dummy variable for gender in the regressions pooling results for men and women in Nicolaou et al. (2018).

23. There are also animal studies with conflicting results; while Zheng and Cohn (2011) find that causally manipulating prenatal testosterone exposure by inactivating androgen or estrogen receptors among mice has the expected outcome on 2D:4D, Huber et al. (2017) find the opposite.

24. There are other null results on 2D:4D and economic preferences (e.g., Parslow et al., 2019; Pearson & Schipper, 2012).
