# Supplementary material for: 2D:4D and Self-Employment: A Preregistered Replication Study in a Large General Population Sample
Source: Entrep Theory Pract. 2021 Jan 5;46(1):21–43. doi: 10.1177/1042258720985478 (PMC13104762; doi:10.1177/1042258720985478)
Supplement: Table S1 - Supplemental material for 2D:4D and Self-Employment: A Preregistered Replication Study in a Large General Population Sample [file sj-pdf-1-etp-10.1177_1042258720985478.pdf]

2D:4D and Self-employment:
A Pre-registered Replication Study in a Large General Population Sample

Supplementary Information

Frank M. Fossen, Levent Neyse, Magnus Johannesson, Anna Dreber

1 Figure

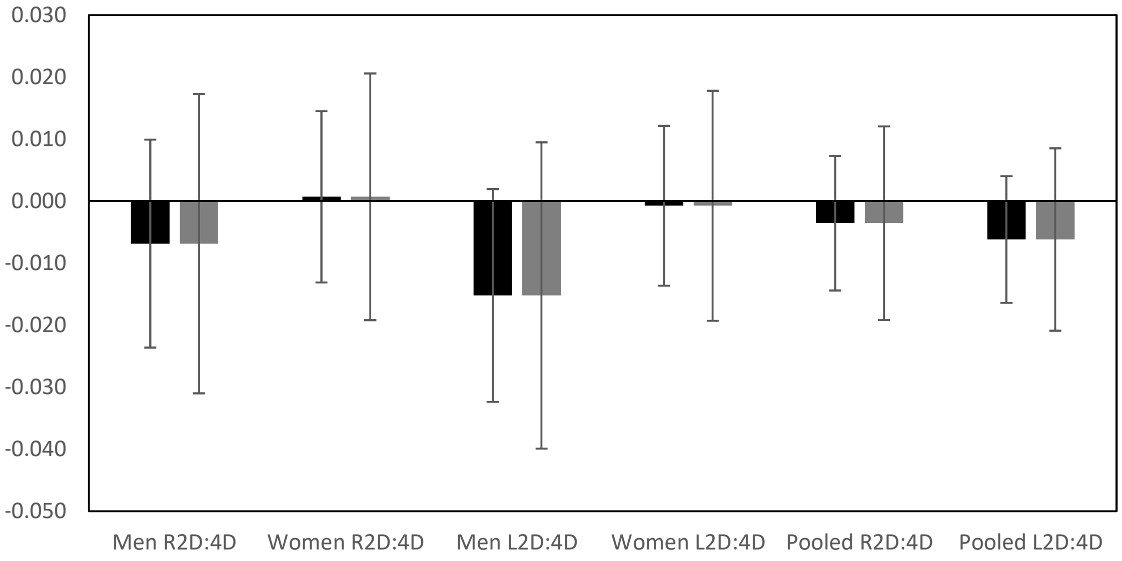

Figure S1: Effect sizes for primary hypotheses estimated by OLS (%95 and %99.5 confidence intervals). The units of the effect sizes are the percentage units changes in self-employment for a one standard deviation change in 2D:4D.

2 Correlation matrices by gender (not pre-registered)

Table S1: Descriptive Statistics and Correlation Matrix for Men

	Self-Employed	R2D:4D	L2D:4D	Mean 2D:4D	Year of Birth	Below Abitur	Abitur or Apprent.	Oth. Hi. Educ.	College	Health	Urban	Gross Pers. Inc.
Panel 1: Descriptive Statistics												
Mean	0.1149	0.9984	0.9981	0.9981	1968.4	0.0497	0.4937	0.1811	0.2756	2.5112	0.5531	3.3733
Std. Dev.	0.3191	0.0483	0.0568	0.0426	14.806	0.2173	0.5002	0.3853	0.4470	0.8845	0.4974	2.6495
Min.	0	0.8235	0.8308	0.8658	1930	0	0	0	0	1	0	0
Max.	1	1.4474	1.4000	1.1977	2000	1	1	1	1	5	1	34
N	1027	1021	1027	1015	1027	1027	1027	1027	1027	1027	1027	1027
Panel 2: Correlation Matrix												
R2D:4D	-0.0422	1.0000										
	0.1796											
L2D:4D	-0.0538	0.3132	1.0000									
	0.0869	0.0000										
Mean 2D:4D	-0.0597	0.7759	0.8422	1.0000								
	0.0575	0.0000	0.0000									
Year of Birth	-0.2035	0.0646	0.0625	0.0782	1.0000							
	0.0000	0.0397	0.0466	0.0127								
Below Abitur	-0.0684	0.0310	0.0501	0.0509	0.1756	1.0000						
	0.0294	0.3232	0.1106	0.1050	0.0000							
Abitur or Apprent.	-0.1060	0.0223	-0.0268	-0.0052	0.0192	-0.2247	1.0000					
	0.0007	0.4781	0.3928	0.8691	0.5402	0.0000						
Oth. Higher Educ.	0.0138	0.0007	-0.0093	-0.0058	-0.0464	-0.1068	-0.4630	1.0000				
	0.6607	0.9820	0.7661	0.8534	0.1397	0.0007	0.0000					
College	0.1397	-0.0405	0.0138	-0.0138	-0.0666	-0.1408	-0.6109	-0.2902	1.0000			
	0.0000	0.1970	0.6607	0.6594	0.0339	0.0000	0.0000	0.0000				
Health	0.0119	0.0124	-0.0229	-0.0082	-0.3223	0.0069	0.0709	0.0581	-0.1324	1.0000		
	0.7047	0.6935	0.4658	0.7943	0.0000	0.8271	0.0240	0.0643	0.0000			
Urban	0.0048	0.0018	-0.0374	-0.0238	-0.0551	-0.0516	-0.0829	-0.0523	0.1625	-0.0111	1.0000	
	0.8780	0.9555	0.2343	0.4483	0.0792	0.1004	0.0083	0.0959	0.0000	0.7238		
Gross Pers. Inc.	0.1235	-0.0316	0.0242	-0.0019	0.0003	-0.1364	-0.2319	-0.0200	0.3423	-0.1826	0.0411	1.0000
	0.0001	0.3145	0.4417	0.9522	0.9924	0.0000	0.0000	0.5239	0.0000	0.0000	0.1907	

Notes: The descriptives for R2D:4D (L2D:4D) are for the n=1021 (n=1027) included in the primary analyses; and the descriptives for the mean 2D:4D are for the n=1015 included in robustness test 1. The descriptives for self-employment and the control variables are for the n=1027 included in the primary analyses for left-hand 2D:4D. Gross personal income is in euro 1000 per month. The correlation matrix shows correlation coefficients and p-values of tests of significant differences from zero below. Here we use all observations from our main estimations with right- and left-hand 2D:4D available (N=1015).

Table S2: Descriptive Statistics and Correlation Matrix for Women

	Self-Employed	R2D:4D	L2D:4D	Mean 2D:4D	Year of Birth	Below Abitur	Abitur or Apprent.	Oth. Hi. Educ.	College	Health	Urban	Gross Pers. Inc.
Panel 1: Descriptive Statistics												
Mean	0.0762	1.0014	1.0050	1.0032	1969.2	0.1036	0.4269	0.2383	0.2312	2.5075	0.5598	1.9622
Std. Dev.	0.2654	0.0492	0.0768	0.0508	14.361	0.3049	0.4949	0.4262	0.4218	0.9081	0.4966	1.5165
Min.	0	0.8002	0.3714	0.6709	1934	0	0	0	0	1	0	0
Max.	1	1.2323	2.2727	1.6513	2001	1	1	1	1	5	1	11
N	1129	1130	1129	1123	1129	1129	1129	1129	1129	1129	1129	1129
Panel 2: Correlation Matrix												
R2D:4D	0.0046	1.0000										
	0.8767											
L2D:4D	-0.0117	0.2726	1.0000									
	0.6959	0.0000										
Mean 2D:4D	-0.0066	0.6887	0.8853	1.0000								
	0.8262	0.0000	0.0000									
Year of Birth	-0.1176	0.0133	0.0153	0.0180	1.0000							
	0.0001	0.6571	0.6076	0.5475								
Below Abitur	-0.0529	-0.0251	-0.0128	-0.0218	-0.0353	1.0000						
	0.0765	0.4006	0.6692	0.4665	0.2371							
Abitur or Apprent.	-0.0017	-0.0032	-0.0066	-0.0065	-0.0442	-0.2927	1.0000					
	0.9536	0.9160	0.8242	0.8272	0.1389	0.0000						
Oth. Higher Educ.	-0.0107	-0.0213	-0.0049	-0.0140	0.0423	-0.1905	-0.4840	1.0000				
	0.7194	0.4753	0.8703	0.6398	0.1564	0.0000	0.0000					
College	0.0511	0.0434	0.0220	0.0375	0.0345	-0.1858	-0.4722	-0.3073	1.0000			
	0.0868	0.1457	0.4623	0.2087	0.2478	0.0000	0.0000	0.0000				
Health	0.0439	0.0338	-0.0075	0.0107	-0.1871	0.0551	0.0572	0.0309	-0.1382	1.0000		
	0.1416	0.2572	0.8011	0.7207	0.0000	0.0650	0.0555	0.3010	0.0000			
Urban	0.0157	-0.0070	-0.0044	-0.0067	0.0473	-0.0004	-0.0534	-0.0753	0.1393	0.0103	1.0000	
	0.5990	0.8135	0.8823	0.8215	0.1132	0.9881	0.0737	0.0116	0.0000	0.7297		
Gross Pers. Inc.	0.0066	-0.0035	-0.0186	-0.0157	0.0284	-0.2093	-0.1963	0.0085	0.3731	-0.1187	0.0877	1.0000
	0.8260	0.9054	0.5339	0.5988	0.3424	0.0000	0.0000	0.7761	0.0000	0.0001	0.0033	

Notes: The descriptives for R2D:4D (L2D:4D) are for the n=1130 (n=1129) included in the primary analyses; and the descriptives for the mean 2D:4D are for the n=1123 included in robustness test 1. The descriptives for self-employment and the control variables are for the n=1129 included in the primary analyses for left-hand 2D:4D. Gross personal income is in euro 1000 per month. The correlation matrix shows correlation coefficients and p-values of tests of significant differences from zero below. Here we use all observations from our main estimations with right- and left-hand 2D:4D available (N=1123).

3 Robustness tests

Table S3: Robustness Test 2a: Corrected Sample

	Men	Women	Men	Women	Both	Both
R2D:4D	-2.405	0.704			-0.824	
	(2.308)	(2.281)			(1.642)	
L2D:4D			-3.324	-0.003		-1.674
			(2.081)	(2.151)		(1.481)
Female					-0.261	-0.232
					(0.161)	(0.161)
Year of Birth	-0.049**	-0.034**	-0.049**	-0.034**	-0.043**	-0.042**
	(0.008)	(0.009)	(0.008)	(0.009)	(0.006)	(0.006)
Below Abitur	-0.985	-0.954	-0.999	-0.961	-0.872	-0.877
	(1.033)	(0.544)	(1.033)	(0.543)	(0.476)	(0.476)
Oth. Higher Educ.	0.336	-0.005	0.336	0.012	0.177	0.188
	(0.284)	(0.298)	(0.285)	(0.298)	(0.205)	(0.205)
College	0.744**	0.451	0.758**	0.501	0.594**	0.616**
	(0.246)	(0.295)	(0.246)	(0.292)	(0.186)	(0.186)
Health	-0.079	0.115	-0.069	0.119	0.023	0.028
	(0.126)	(0.125)	(0.125)	(0.125)	(0.089)	(0.088)
Urban	-0.148	0.101	-0.166	0.127	-0.038	-0.032
	(0.209)	(0.235)	(0.209)	(0.233)	(0.155)	(0.155)
Gross Pers. Inc.	0.080*	-0.037	0.083*	-0.044	0.068*	0.067*
	(0.033)	(0.080)	(0.033)	(0.079)	(0.029)	(0.029)
Constant	97.112**	63.281**	96.736**	64.015**	82.278**	82.463**
	(16.418)	(17.530)	(16.358)	(17.424)	(11.935)	(11.894)
R2D:4D (ME)	-0.227	0.048			-0.067	
	(0.218)	(0.155)			(0.133)	
L2D:4D (ME)			-0.311	-0.000		-0.136
			(0.195)	(0.148)		(0.120)
Observations	1021	1130	1027	1129	2151	2156
Pseudo R^2	0.100	0.041	0.101	0.042	0.074	0.075
χ^2	72.81	24.62	73.94	25.69	99.63	100.64
p-value	0.000	0.002	0.000	0.001	0.000	0.000

Logit regressions; standard errors in parentheses; * $p < 0.05$, ** $p < 0.005$. (ME) shows marginal effects. Right and left 2D:4D's are represented by R2D:4D and L2D:4D respectively.

Table S4: Robustness Test 2b: Corrected Sample with Mean 2D:4D

	Men	Women	Both
Mean 2D:4D	-4.327	-0.481	-2.396
	(2.653)	(2.715)	(1.900)
Female			-0.248
			(0.161)
Year of Birth	-0.049**	-0.034**	-0.043**
	(0.008)	(0.009)	(0.006)
Below Abitur	-0.995	-0.953	-0.869
	(1.034)	(0.544)	(0.476)
Oth. Higher Educ.	0.335	-0.001	0.181
	(0.285)	(0.298)	(0.205)
College	0.744**	0.453	0.594**
	(0.246)	(0.295)	(0.186)
Health	-0.081	0.128	0.027
	(0.126)	(0.125)	(0.089)
Urban	-0.162	0.100	-0.044
	(0.209)	(0.234)	(0.155)
Gross Pers. Inc.	0.081*	-0.034	0.068*
	(0.033)	(0.079)	(0.029)
Constant	98.407**	64.245**	83.419**
	(16.403)	(17.510)	(11.933)
Mean 2D:4D (ME)	-0.409	-0.033	-0.195
	(0.251)	(0.186)	(0.155)
Observations	1015	1123	2138
Pseudo R^2	0.102	0.041	0.075
χ^2	74.32	24.87	101.25
p-value	0.000	0.002	0.000

Logit regressions; standard errors in parentheses;

* $p < 0.05$, ** $p < 0.005$. (ME) shows marginal effects.

Table S5: Robustness Test 3a: Restricted Sample

	Men	Women	Men	Women	Both	Both
R2D:4D	-1.705	0.582			-0.613	
	(2.269)	(2.357)			(1.625)	
L2D:4D			-2.728	-0.445		-1.603
			(2.009)	(2.074)		(1.436)
Female					-0.261	-0.231
					(0.161)	(0.161)
Year of Birth	-0.050**	-0.034**	-0.049**	-0.034**	-0.043**	-0.042**
	(0.008)	(0.009)	(0.008)	(0.009)	(0.006)	(0.006)
Below Abitur	-0.970	-0.956	-0.947	-0.966	-0.868	-0.874
	(1.033)	(0.544)	(1.033)	(0.543)	(0.476)	(0.476)
Oth. Higher Educ.	0.348	-0.005	0.333	0.003	0.182	0.182
	(0.284)	(0.298)	(0.285)	(0.299)	(0.205)	(0.205)
College	0.744**	0.460	0.754**	0.454	0.597**	0.598**
	(0.246)	(0.295)	(0.246)	(0.296)	(0.186)	(0.186)
Health	-0.079	0.114	-0.068	0.127	0.022	0.031
	(0.126)	(0.125)	(0.125)	(0.126)	(0.089)	(0.089)
Urban	-0.150	0.104	-0.162	0.096	-0.037	-0.046
	(0.209)	(0.235)	(0.209)	(0.234)	(0.155)	(0.155)
Gross Pers. Inc.	0.080*	-0.038	0.082*	-0.030	0.067*	0.069*
	(0.033)	(0.080)	(0.033)	(0.079)	(0.029)	(0.029)
Constant	96.748**	63.844**	96.088**	63.711**	82.427**	82.081**
	(16.412)	(17.550)	(16.345)	(17.482)	(11.941)	(11.907)
R2D:4D (ME)	-0.161	0.040			-0.050	
	(0.214)	(0.160)			(0.132)	
L2D:4D (ME)			-0.257	-0.031		-0.131
			(0.189)	(0.143)		(0.117)
Observations	1019	1129	1021	1116	2148	2137
Pseudo R^2	0.099	0.041	0.099	0.041	0.074	0.074
χ^2	72.27	24.82	72.53	24.80	99.80	99.62
p-value	0.000	0.002	0.000	0.002	0.000	0.000

Logit regressions; standard errors in parentheses; * $p < 0.05$, ** $p < 0.005$. (ME) shows marginal effects. Right and left 2D:4D's are represented by R2D:4D and L2D:4D respectively.

Table S6: Robustness Test 3b: Restricted Sample with Mean 2D:4D

	Men	Women	Both
Mean 2D:4D	-3.304	0.096	-1.659
	(2.582)	(2.715)	(1.859)
Female			-0.241
			(0.162)
Year of Birth	-0.049**	-0.034**	-0.043**
	(0.008)	(0.009)	(0.006)
Below Abitur	-0.929	-0.964	-0.867
	(1.034)	(0.544)	(0.477)
Oth. Higher Educ.	0.340	-0.009	0.179
	(0.285)	(0.298)	(0.205)
College	0.739**	0.456	0.593**
	(0.246)	(0.296)	(0.186)
Health	-0.079	0.125	0.026
	(0.126)	(0.126)	(0.089)
Urban	-0.161	0.092	-0.049
	(0.209)	(0.235)	(0.155)
Gross Pers. Inc.	0.081*	-0.029	0.068*
	(0.033)	(0.079)	(0.029)
Constant	97.544**	64.303**	83.004**
	(16.390)	(17.521)	(11.930)
Mean 2D:4D (ME)	-0.315	0.007	-0.136
	(0.246)	(0.188)	(0.153)
Observations	1007	1110	2117
Pseudo R^2	0.100	0.042	0.075
χ^2	72.61	25.17	100.30
p-value	0.000	0.001	0.000

Logit regressions; standard errors in parentheses;
* $p < 0.05$, ** $p < 0.005$. (ME) shows marginal effects.

Table S7: Robustness Test 4a: Primary Hypotheses (OLS)

	Men	Women	Men	Women	Both	Both
R2D:4D	-0.142	0.014			-0.073	
	(0.178)	(0.144)			(0.114)	
L2D:4D			-0.268	-0.010		-0.091
			(0.155)	(0.086)		(0.077)
Female					-0.024	-0.023
					(0.014)	(0.014)
Year of Birth	-0.004**	-0.002**	-0.004**	-0.002**	-0.003**	-0.003**
	(0.001)	(0.001)	(0.001)	(0.001)	(0.000)	(0.000)
Below Abitur	-0.003	-0.045*	-0.003	-0.046*	-0.028	-0.028
	(0.027)	(0.022)	(0.026)	(0.022)	(0.017)	(0.017)
Oth. Higher Educ.	0.031	0.001	0.031	0.002	0.015	0.016
	(0.026)	(0.020)	(0.026)	(0.020)	(0.016)	(0.016)
College	0.078*	0.035	0.079*	0.039	0.055**	0.058**
	(0.029)	(0.025)	(0.029)	(0.025)	(0.019)	(0.019)
Health	-0.009	0.008	-0.008	0.008	0.002	0.002
	(0.013)	(0.009)	(0.013)	(0.010)	(0.008)	(0.008)
Urban	-0.016	0.008	-0.018	0.010	-0.003	-0.002
	(0.020)	(0.016)	(0.020)	(0.016)	(0.013)	(0.013)
Gross Pers. Inc.	0.010	-0.004	0.010	-0.004	0.007	0.007
	(0.006)	(0.009)	(0.006)	(0.009)	(0.005)	(0.005)
Constant	8.766**	4.205**	8.747**	4.291**	6.353**	6.349**
	(1.448)	(1.071)	(1.442)	(1.072)	(0.886)	(0.884)
Observations	1021	1130	1027	1129	2151	2156
Adjusted R^2	0.060	0.013	0.061	0.014	0.040	0.040

OLS regressions; robust standard errors in parentheses; * $p < 0.05$, ** $p < 0.005$.

Right and left 2D:4D's are represented by R2D:4D and L2D:4D respectively.

Table S8: Robustness Test 4b: Mean 2D:4D (OLS)

	Men	Women	Both
Mean 2D:4D	-0.323	-0.043	-0.155
	(0.211)	(0.136)	(0.117)
Female			-0.024
			(0.014)
Year of Birth	-0.004**	-0.002**	-0.003**
	(0.001)	(0.001)	(0.000)
Below Abitur	-0.002	-0.045*	-0.028
	(0.027)	(0.022)	(0.017)
Oth. Higher Educ.	0.031	0.001	0.016
	(0.026)	(0.020)	(0.016)
College	0.078*	0.035	0.055**
	(0.029)	(0.025)	(0.019)
Health	-0.009	0.009	0.002
	(0.013)	(0.010)	(0.008)
Urban	-0.017	0.008	-0.003
	(0.020)	(0.016)	(0.013)
Gross Pers. Inc.	0.010	-0.003	0.007
	(0.006)	(0.009)	(0.005)
Constant	8.955**	4.292**	6.461**
	(1.463)	(1.080)	(0.894)
Observations	1015	1123	2138
Adjusted R^2	0.062	0.014	0.041

OLS regressions; robust standard errors in parentheses;
* $p < 0.05$, ** $p < 0.005$.

Table S9: Robustness Test 4c: Corrected Sample (OLS)

	Men	Women	Men	Women	Both	Both
R2D:4D	-0.217	0.040			-0.079	
	(0.202)	(0.148)			(0.121)	
L2D:4D			-0.314	-0.009		-0.140
			(0.179)	(0.171)		(0.125)
Female					-0.024	-0.022
					(0.014)	(0.014)
Year of Birth	-0.004**	-0.002**	-0.004**	-0.002**	-0.003**	-0.003**
	(0.001)	(0.001)	(0.001)	(0.001)	(0.000)	(0.000)
Below Abitur	-0.005	-0.045*	-0.006	-0.046*	-0.028	-0.028
	(0.027)	(0.022)	(0.027)	(0.022)	(0.017)	(0.017)
Oth. Higher Educ.	0.031	0.001	0.030	0.002	0.015	0.016
	(0.026)	(0.020)	(0.026)	(0.020)	(0.016)	(0.016)
College	0.078*	0.034	0.080*	0.039	0.055**	0.058**
	(0.029)	(0.025)	(0.029)	(0.025)	(0.019)	(0.019)
Health	-0.009	0.008	-0.009	0.008	0.002	0.002
	(0.013)	(0.009)	(0.013)	(0.010)	(0.008)	(0.008)
Urban	-0.017	0.008	-0.018	0.010	-0.003	-0.002
	(0.020)	(0.016)	(0.020)	(0.016)	(0.013)	(0.013)
Gross Pers. Inc.	0.010	-0.004	0.010	-0.004	0.007	0.007
	(0.006)	(0.009)	(0.006)	(0.009)	(0.005)	(0.005)
Constant	8.829**	4.184**	8.788**	4.290**	6.361**	6.382**
	(1.448)	(1.070)	(1.436)	(1.077)	(0.885)	(0.885)
Observations	1021	1130	1027	1129	2151	2156
Adjusted R^2	0.061	0.013	0.061	0.014	0.040	0.041

OLS regressions; robust standard errors in parentheses; * $p < 0.05$, ** $p < 0.005$.

Right and left 2D:4D's are represented by R2D:4D and L2D:4D respectively.

Table S10: Robustness Test 4d: Corrected Sample with Mean 2D:4D (OLS)

	Men	Women	Both
Mean 2D:4D	-0.401	-0.049	-0.203
	(0.232)	(0.186)	(0.146)
Female			-0.024
			(0.014)
Year of Birth	-0.004**	-0.002**	-0.003**
	(0.001)	(0.001)	(0.000)
Below Abitur	-0.006	-0.045*	-0.028
	(0.027)	(0.022)	(0.017)
Oth. Higher Educ.	0.031	0.001	0.016
	(0.026)	(0.020)	(0.016)
College	0.078*	0.035	0.056**
	(0.029)	(0.025)	(0.019)
Health	-0.010	0.009	0.002
	(0.013)	(0.010)	(0.008)
Urban	-0.018	0.008	-0.003
	(0.020)	(0.016)	(0.013)
Gross Pers. Inc.	0.010	-0.003	0.007
	(0.006)	(0.009)	(0.005)
Constant	9.028**	4.294**	6.497**
	(1.458)	(1.083)	(0.893)
Observations	1015	1123	2138
Adjusted R^2	0.062	0.014	0.041

OLS regressions; robust standard errors in parentheses;
* $p < 0.05$, ** $p < 0.005$.

Table S11: Robustness Test 4e: Restricted Sample (OLS)

	Men	Women	Men	Women	Both	Both
R2D:4D	-0.145	0.027			-0.061	
	(0.197)	(0.146)			(0.120)	
L2D:4D			-0.263	-0.042		-0.136
			(0.185)	(0.153)		(0.118)
Female					-0.024	-0.022
					(0.014)	(0.014)
Year of Birth	-0.004**	-0.002**	-0.004**	-0.002**	-0.003**	-0.003**
	(0.001)	(0.001)	(0.001)	(0.001)	(0.000)	(0.000)
Below Abitur	-0.005	-0.045*	-0.004	-0.046*	-0.028	-0.028
	(0.027)	(0.022)	(0.027)	(0.022)	(0.017)	(0.017)
Oth. Higher Educ.	0.032	0.001	0.030	0.001	0.016	0.016
	(0.026)	(0.020)	(0.026)	(0.021)	(0.016)	(0.016)
College	0.078*	0.035	0.079*	0.035	0.056**	0.056**
	(0.029)	(0.025)	(0.029)	(0.025)	(0.019)	(0.019)
Health	-0.009	0.008	-0.008	0.009	0.002	0.003
	(0.013)	(0.009)	(0.013)	(0.010)	(0.008)	(0.008)
Urban	-0.017	0.008	-0.018	0.008	-0.003	-0.003
	(0.020)	(0.016)	(0.020)	(0.016)	(0.013)	(0.013)
Gross Pers. Inc.	0.010	-0.004	0.010	-0.003	0.007	0.007
	(0.006)	(0.009)	(0.006)	(0.009)	(0.005)	(0.005)
Constant	8.804**	4.226**	8.769**	4.269**	6.374**	6.374**
	(1.453)	(1.072)	(1.451)	(1.084)	(0.888)	(0.892)
Observations	1019	1129	1021	1116	2148	2137
Adjusted R^2	0.060	0.013	0.060	0.014	0.040	0.040

OLS regressions; robust standard errors in parentheses; * $p < 0.05$, ** $p < 0.005$.

Right and left 2D:4D's are represented by R2D:4D and L2D:4D respectively.

Table S12: Robustness Test 4f: Restricted Sample with Mean 2D:4D (OLS)

	Men	Women	Both
Mean 2D:4D	-0.300	-0.014	-0.147
	(0.231)	(0.194)	(0.149)
Female			-0.023
			(0.014)
Year of Birth	-0.004**	-0.002**	-0.003**
	(0.001)	(0.001)	(0.000)
Below Abitur	-0.004	-0.046*	-0.028
	(0.028)	(0.022)	(0.017)
Oth. Higher Educ.	0.032	0.001	0.016
	(0.027)	(0.021)	(0.016)
College	0.078*	0.036	0.056**
	(0.029)	(0.026)	(0.019)
Health	-0.010	0.009	0.002
	(0.013)	(0.010)	(0.008)
Urban	-0.018	0.007	-0.004
	(0.020)	(0.016)	(0.013)
Gross Pers. Inc.	0.010	-0.003	0.007
	(0.006)	(0.009)	(0.005)
Constant	8.995**	4.326**	6.510**
	(1.474)	(1.092)	(0.903)
Observations	1007	1110	2117
Adjusted R^2	0.061	0.014	0.041

OLS regressions; robust standard errors in parentheses;
* $p < 0.05$, ** $p < 0.005$.

Table S13: Robustness Test 5a: Primary Hypotheses without Self-employed Farmers

	Men	Women	Men	Women	Both	Both
R2D:4D	-1.830	-0.311			-1.124	
	(2.268)	(2.356)			(1.623)	
L2D:4D			-2.901	-0.575		-1.446
			(1.978)	(1.515)		(1.205)
Female					-0.270	-0.245
					(0.164)	(0.164)
Year of Birth	-0.050**	-0.036**	-0.049**	-0.037**	-0.044**	-0.044**
	(0.008)	(0.009)	(0.008)	(0.009)	(0.006)	(0.006)
Below Abitur	-0.953	-0.904	-0.941	-0.905	-0.834	-0.836
	(1.034)	(0.546)	(1.033)	(0.546)	(0.477)	(0.477)
Oth. Higher Educ.	0.276	-0.011	0.274	0.009	0.143	0.152
	(0.293)	(0.306)	(0.293)	(0.306)	(0.211)	(0.211)
College	0.745**	0.445	0.757**	0.498	0.600**	0.622**
	(0.248)	(0.303)	(0.248)	(0.299)	(0.189)	(0.188)
Health	-0.086	0.138	-0.075	0.138	0.028	0.032
	(0.128)	(0.128)	(0.127)	(0.127)	(0.090)	(0.090)
Urban	-0.091	0.108	-0.106	0.138	0.001	0.009
	(0.213)	(0.240)	(0.213)	(0.239)	(0.159)	(0.158)
Gross Pers. Inc.	0.071*	-0.012	0.074*	-0.020	0.065*	0.064*
	(0.033)	(0.080)	(0.033)	(0.079)	(0.030)	(0.030)
Constant	97.155**	68.920**	96.679**	69.393**	84.955**	84.732**
	(16.657)	(18.126)	(16.580)	(17.991)	(12.221)	(12.166)
R2D:4D (ME)	-0.168	-0.020			-0.088	
	(0.208)	(0.154)			(0.127)	
L2D:4D (ME)			-0.264	-0.038		-0.113
			(0.180)	(0.100)		(0.095)
Observations	1016	1126	1022	1125	2142	2147
Pseudo R^2	0.097	0.045	0.098	0.046	0.076	0.076
χ^2	68.50	25.92	69.68	27.21	98.83	99.88
p-value	0.000	0.001	0.000	0.001	0.000	0.000

Logit regressions; standard errors in parentheses; * $p < 0.05$, ** $p < 0.005$. (ME) shows marginal effects. Right and left 2D:4D's are represented by R2D:4D and L2D:4D respectively.

Table S14: Robustness Test 5b: Mean 2D:4D without Self-employed Farmers

	Men	Women	Both
Mean 2D:4D	-3.617	-1.395	-2.393
	(2.566)	(2.322)	(1.718)
Female			-0.260
			(0.164)
Year of Birth	-0.049**	-0.036**	-0.044**
	(0.008)	(0.009)	(0.006)
Below Abitur	-0.938	-0.899	-0.826
	(1.034)	(0.546)	(0.477)
Oth. Higher Educ.	0.274	-0.005	0.146
	(0.293)	(0.306)	(0.211)
College	0.742**	0.449	0.600**
	(0.248)	(0.302)	(0.189)
Health	-0.088	0.148	0.031
	(0.128)	(0.128)	(0.090)
Urban	-0.101	0.107	-0.004
	(0.213)	(0.240)	(0.159)
Gross Pers. Inc.	0.072*	-0.010	0.065*
	(0.033)	(0.080)	(0.030)
Constant	98.174**	69.910**	85.835**
	(16.622)	(18.095)	(12.206)
Mean 2D:4D (ME)	-0.332	-0.091	-0.188
	(0.236)	(0.152)	(0.135)
Observations	1010	1119	2129
Pseudo R^2	0.099	0.046	0.077
χ^2	69.79	26.64	100.60
p-value	0.000	0.001	0.000

Logit regressions; standard errors in parentheses;
* $p < 0.05$, ** $p < 0.005$. (ME) shows marginal effects.

Table S15: Robustness Test 5c: Corrected Sample without Self-employed Farmers

	Men	Women	Men	Women	Both	Both
R2D:4D	-2.396	0.055			-1.164	
	(2.344)	(2.346)			(1.674)	
L2D:4D			-3.327	-1.181		-2.274
			(2.114)	(2.201)		(1.515)
Female					-0.270	-0.237
					(0.164)	(0.164)
Year of Birth	-0.050**	-0.036**	-0.049**	-0.036**	-0.044**	-0.044**
	(0.008)	(0.009)	(0.008)	(0.009)	(0.006)	(0.006)
Below Abitur	-0.962	-0.901	-0.975	-0.900	-0.836	-0.835
	(1.034)	(0.546)	(1.033)	(0.546)	(0.477)	(0.477)
Oth. Higher Educ.	0.272	-0.010	0.274	0.013	0.142	0.156
	(0.293)	(0.306)	(0.293)	(0.306)	(0.211)	(0.211)
College	0.747**	0.444	0.759**	0.501	0.601**	0.625**
	(0.248)	(0.303)	(0.248)	(0.299)	(0.189)	(0.189)
Health	-0.087	0.137	-0.075	0.138	0.028	0.032
	(0.128)	(0.128)	(0.127)	(0.128)	(0.090)	(0.090)
Urban	-0.091	0.109	-0.109	0.138	0.000	0.006
	(0.213)	(0.240)	(0.214)	(0.239)	(0.159)	(0.158)
Gross Pers. Inc.	0.071*	-0.011	0.075*	-0.020	0.065*	0.065*
	(0.033)	(0.080)	(0.033)	(0.079)	(0.030)	(0.030)
Constant	97.545**	68.685**	97.127**	69.797**	84.964**	85.317**
	(16.659)	(18.118)	(16.599)	(18.026)	(12.219)	(12.181)
R2D:4D (ME)	-0.219	0.004			-0.091	
	(0.215)	(0.153)			(0.131)	
L2D:4D (ME)			-0.303	-0.078		-0.178
			(0.192)	(0.145)		(0.119)
Observations	1016	1126	1022	1125	2142	2147
Pseudo R^2	0.097	0.044	0.098	0.047	0.076	0.077
χ^2	68.89	25.90	69.99	27.35	98.83	100.66
p-value	0.000	0.001	0.000	0.001	0.000	0.000

Logit regressions; standard errors in parentheses; * $p < 0.05$, ** $p < 0.005$. (ME) shows marginal effects. Right and left 2D:4D's are represented by R2D:4D and L2D:4D respectively.

Table S16: Robustness Test 5d: Corrected Sample with Mean 2D:4D without Self-employed Farmers

	Men	Women	Both
Mean 2D:4D	-4.333	-1.937	-3.142
	(2.697)	(2.793)	(1.940)
Female			-0.255
			(0.164)
Year of Birth	-0.049**	-0.036**	-0.044**
	(0.008)	(0.009)	(0.006)
Below Abitur	-0.973	-0.897	-0.831
	(1.034)	(0.546)	(0.477)
Oth. Higher Educ.	0.273	-0.002	0.148
	(0.294)	(0.306)	(0.211)
College	0.745**	0.450	0.602**
	(0.249)	(0.302)	(0.189)
Health	-0.089	0.150	0.032
	(0.128)	(0.128)	(0.091)
Urban	-0.104	0.107	-0.007
	(0.214)	(0.240)	(0.159)
Gross Pers. Inc.	0.073*	-0.010	0.066*
	(0.033)	(0.080)	(0.030)
Constant	98.768**	70.184**	86.335**
	(16.638)	(18.108)	(12.217)
Mean 2D:4D (ME)	-0.398	-0.127	-0.247
	(0.248)	(0.183)	(0.152)
Observations	1010	1119	2129
Pseudo R^2	0.099	0.046	0.078
χ^2	70.37	26.75	101.25
p-value	0.000	0.001	0.000

Logit regressions; standard errors in parentheses;

* $p < 0.05$, ** $p < 0.005$. (ME) shows marginal effects.

Table S17: Robustness Test 5e: Restricted Sample without Self-employed Farmers

	Men	Women	Men	Women	Both	Both
R2D:4D	-1.666	-0.091			-0.924	
	(2.305)	(2.406)			(1.656)	
L2D:4D			-2.692	-1.562		-2.149
			(2.041)	(2.128)		(1.468)
Female					-0.270	-0.237
					(0.164)	(0.165)
Year of Birth	-0.050**	-0.037**	-0.049**	-0.036**	-0.044**	-0.043**
	(0.008)	(0.009)	(0.008)	(0.009)	(0.006)	(0.006)
Below Abitur	-0.947	-0.904	-0.925	-0.907	-0.831	-0.832
	(1.034)	(0.546)	(1.034)	(0.546)	(0.477)	(0.477)
Oth. Higher Educ.	0.283	-0.010	0.271	0.006	0.148	0.151
	(0.293)	(0.306)	(0.293)	(0.307)	(0.211)	(0.211)
College	0.746**	0.453	0.754**	0.449	0.603**	0.606**
	(0.248)	(0.303)	(0.248)	(0.303)	(0.189)	(0.189)
Health	-0.086	0.136	-0.075	0.146	0.027	0.035
	(0.128)	(0.128)	(0.127)	(0.128)	(0.090)	(0.090)
Urban	-0.093	0.112	-0.105	0.103	0.001	-0.009
	(0.213)	(0.240)	(0.213)	(0.240)	(0.159)	(0.159)
Gross Pers. Inc.	0.071*	-0.013	0.074*	-0.006	0.064*	0.067*
	(0.033)	(0.080)	(0.033)	(0.080)	(0.030)	(0.030)
Constant	97.172**	69.245**	96.495**	69.470**	85.089**	84.886**
	(16.655)	(18.140)	(16.588)	(18.091)	(12.226)	(12.193)
R2D:4D (ME)	-0.153	-0.006			-0.072	
	(0.211)	(0.157)			(0.130)	
L2D:4D (ME)			-0.247	-0.103		-0.169
			(0.187)	(0.140)		(0.115)
Observations	1014	1125	1016	1112	2139	2128
Pseudo R^2	0.096	0.045	0.097	0.046	0.076	0.077
χ^2	68.32	26.14	68.59	26.73	98.94	99.51
p-value	0.000	0.001	0.000	0.001	0.000	0.000

Logit regressions; standard errors in parentheses; * $p < 0.05$, ** $p < 0.005$. (ME) shows marginal effects. Right and left 2D:4D's are represented by R2D:4D and L2D:4D respectively.

Table S18: Robustness Test 5f: Restricted Sample with Mean 2D:4D without Self-employed Farmers

	Men	Women	Both
Mean 2D:4D	-3.261	-1.301	-2.321
	(2.625)	(2.779)	(1.899)
Female			-0.248
			(0.165)
Year of Birth	-0.049**	-0.037**	-0.044**
	(0.008)	(0.009)	(0.006)
Below Abitur	-0.908	-0.909	-0.828
	(1.035)	(0.546)	(0.478)
Oth. Higher Educ.	0.277	-0.009	0.146
	(0.293)	(0.306)	(0.211)
College	0.740**	0.451	0.601**
	(0.249)	(0.303)	(0.189)
Health	-0.087	0.147	0.031
	(0.128)	(0.128)	(0.091)
Urban	-0.103	0.097	-0.012
	(0.213)	(0.241)	(0.159)
Gross Pers. Inc.	0.072*	-0.004	0.066*
	(0.033)	(0.080)	(0.030)
Constant	97.910**	70.118**	85.829**
	(16.628)	(18.111)	(12.212)
Mean 2D:4D (ME)	-0.302	-0.086	-0.184
	(0.243)	(0.184)	(0.150)
Observations	1002	1106	2108
Pseudo R^2	0.097	0.046	0.077
χ^2	68.66	26.84	99.95
p-value	0.000	0.001	0.000

Logit regressions; standard errors in parentheses;

* $p < 0.05$, ** $p < 0.005$. (ME) shows marginal effects.

Table S19: Robustness Test 5g: Primary Hypotheses without Self-employed Farmers (OLS)

	Men	Women	Men	Women	Both	Both
R2D:4D	-0.139	-0.032			-0.096	
	(0.177)	(0.141)			(0.112)	
L2D:4D			-0.260	-0.045		-0.114
			(0.154)	(0.083)		(0.076)
Female					-0.025	-0.023
					(0.013)	(0.013)
Year of Birth	-0.004**	-0.002**	-0.004**	-0.002**	-0.003**	-0.003**
	(0.001)	(0.001)	(0.001)	(0.001)	(0.000)	(0.000)
Below Abitur	-0.004	-0.041	-0.004	-0.041	-0.026	-0.026
	(0.027)	(0.022)	(0.026)	(0.021)	(0.017)	(0.016)
Oth. Higher Educ.	0.025	0.000	0.024	0.002	0.013	0.013
	(0.026)	(0.020)	(0.026)	(0.020)	(0.016)	(0.016)
College	0.077*	0.033	0.078*	0.037	0.055**	0.057**
	(0.029)	(0.025)	(0.029)	(0.025)	(0.019)	(0.019)
Health	-0.010	0.009	-0.009	0.009	0.002	0.002
	(0.013)	(0.009)	(0.013)	(0.009)	(0.008)	(0.008)
Urban	-0.010	0.008	-0.012	0.010	0.001	0.001
	(0.020)	(0.016)	(0.020)	(0.016)	(0.012)	(0.012)
Gross Pers. Inc.	0.008	-0.002	0.008	-0.002	0.006	0.006
	(0.006)	(0.008)	(0.006)	(0.008)	(0.005)	(0.005)
Constant	8.563**	4.317**	8.543**	4.396**	6.302**	6.301**
	(1.448)	(1.062)	(1.442)	(1.061)	(0.882)	(0.879)
Observations	1016	1126	1022	1125	2142	2147
Adjusted R^2	0.056	0.014	0.057	0.016	0.040	0.040

OLS regressions; robust standard errors in parentheses; * $p < 0.05$, ** $p < 0.005$.

Right and left 2D:4D's are represented by R2D:4D and L2D:4D respectively.

Table S20: Robustness Test 5h: Mean 2D:4D without Self-employed Farmers (OLS)

	Men	Women	Both
Mean 2D:4D	-0.316	-0.105	-0.193
	(0.208)	(0.131)	(0.115)
Female			-0.024
			(0.014)
Year of Birth	-0.004**	-0.002**	-0.003**
	(0.001)	(0.001)	(0.000)
Below Abitur	-0.003	-0.041	-0.025
	(0.027)	(0.022)	(0.017)
Oth. Higher Educ.	0.025	0.001	0.013
	(0.026)	(0.020)	(0.016)
College	0.077*	0.033	0.055**
	(0.029)	(0.025)	(0.019)
Health	-0.010	0.010	0.002
	(0.013)	(0.009)	(0.008)
Urban	-0.011	0.008	0.000
	(0.020)	(0.016)	(0.013)
Gross Pers. Inc.	0.008	-0.002	0.006
	(0.006)	(0.009)	(0.005)
Constant	8.747**	4.419**	6.419**
	(1.463)	(1.069)	(0.889)
Observations	1010	1119	2129
Adjusted R^2	0.058	0.015	0.041

OLS regressions; robust standard errors in parentheses;
* $p < 0.05$, ** $p < 0.005$.

Table S21: Robustness Test 5i: Corrected Sample without Self-employed Farmers (OLS)

	Men	Women	Men	Women	Both	Both
R2D:4D	-0.216	-0.008			-0.106	
	(0.201)	(0.144)			(0.120)	
L2D:4D			-0.309	-0.088		-0.184
			(0.177)	(0.168)		(0.122)
Female					-0.025	-0.022
					(0.013)	(0.013)
Year of Birth	-0.004**	-0.002**	-0.004**	-0.002**	-0.003**	-0.003**
	(0.001)	(0.001)	(0.001)	(0.001)	(0.000)	(0.000)
Below Abitur	-0.006	-0.041	-0.007	-0.041	-0.026	-0.026
	(0.027)	(0.022)	(0.026)	(0.021)	(0.017)	(0.017)
Oth. Higher Educ.	0.024	0.000	0.024	0.002	0.012	0.013
	(0.026)	(0.020)	(0.026)	(0.020)	(0.016)	(0.016)
College	0.077*	0.033	0.079*	0.037	0.055**	0.057**
	(0.029)	(0.025)	(0.029)	(0.025)	(0.019)	(0.019)
Health	-0.010	0.009	-0.009	0.009	0.002	0.002
	(0.013)	(0.009)	(0.013)	(0.009)	(0.008)	(0.008)
Urban	-0.011	0.008	-0.012	0.010	0.000	0.001
	(0.020)	(0.016)	(0.020)	(0.016)	(0.012)	(0.012)
Gross Pers. Inc.	0.008	-0.002	0.008	-0.002	0.006	0.006
	(0.006)	(0.008)	(0.006)	(0.008)	(0.005)	(0.005)
Constant	8.626**	4.298**	8.584**	4.425**	6.313**	6.347**
	(1.448)	(1.061)	(1.436)	(1.064)	(0.881)	(0.880)
Observations	1016	1126	1022	1125	2142	2147
Adjusted R^2	0.057	0.014	0.058	0.016	0.040	0.041

OLS regressions; robust standard errors in parentheses; * $p < 0.05$, ** $p < 0.005$.

Right and left 2D:4D's are represented by R2D:4D and L2D:4D respectively.

Table S22: Robustness Test 5j: Corrected Sample with Mean 2D:4D without Self-employed Farmers (OLS)

	Men	Women	Both
Mean 2D:4D	-0.396	-0.145	-0.257
	(0.230)	(0.180)	(0.143)
Female			-0.024
			(0.014)
Year of Birth	-0.004**	-0.002**	-0.003**
	(0.001)	(0.001)	(0.000)
Below Abitur	-0.006	-0.041	-0.026
	(0.027)	(0.022)	(0.017)
Oth. Higher Educ.	0.025	0.001	0.013
	(0.026)	(0.020)	(0.016)
College	0.077*	0.033	0.055**
	(0.029)	(0.025)	(0.019)
Health	-0.010	0.010	0.002
	(0.013)	(0.009)	(0.008)
Urban	-0.012	0.008	0.000
	(0.020)	(0.016)	(0.013)
Gross Pers. Inc.	0.008	-0.002	0.006
	(0.006)	(0.009)	(0.005)
Constant	8.820**	4.445**	6.467**
	(1.458)	(1.071)	(0.889)
Observations	1010	1119	2129
Adjusted R^2	0.059	0.015	0.041

OLS regressions; robust standard errors in parentheses;
* $p < 0.05$, ** $p < 0.005$.

Table S23: Robustness Test 5k: Restricted Sample without Self-employed Farmers (OLS)

	Men	Women	Men	Women	Both	Both
R2D:4D	-0.142	-0.020			-0.085	
	(0.196)	(0.143)			(0.119)	
L2D:4D			-0.257	-0.115		-0.175
			(0.184)	(0.149)		(0.116)
Female					-0.025	-0.022
					(0.013)	(0.014)
Year of Birth	-0.004**	-0.002**	-0.004**	-0.002**	-0.003**	-0.003**
	(0.001)	(0.001)	(0.001)	(0.001)	(0.000)	(0.000)
Below Abitur	-0.006	-0.041	-0.005	-0.041	-0.026	-0.026
	(0.027)	(0.022)	(0.027)	(0.022)	(0.017)	(0.017)
Oth. Higher Educ.	0.026	0.000	0.024	0.002	0.013	0.013
	(0.026)	(0.020)	(0.026)	(0.020)	(0.016)	(0.016)
College	0.077*	0.033	0.078*	0.034	0.055**	0.055**
	(0.029)	(0.025)	(0.029)	(0.025)	(0.019)	(0.019)
Health	-0.010	0.009	-0.009	0.010	0.002	0.003
	(0.013)	(0.009)	(0.013)	(0.010)	(0.008)	(0.008)
Urban	-0.011	0.008	-0.012	0.008	0.000	-0.000
	(0.020)	(0.016)	(0.020)	(0.016)	(0.012)	(0.013)
Gross Pers. Inc.	0.008	-0.002	0.008	-0.001	0.006	0.007
	(0.006)	(0.008)	(0.006)	(0.009)	(0.005)	(0.005)
Constant	8.599**	4.336**	8.566**	4.402**	6.323**	6.337**
	(1.454)	(1.063)	(1.451)	(1.072)	(0.885)	(0.887)
Observations	1014	1125	1016	1112	2139	2128
Adjusted R^2	0.057	0.015	0.057	0.015	0.040	0.041

OLS regressions; robust standard errors in parentheses; * $p < 0.05$, ** $p < 0.005$.

Right and left 2D:4D's are represented by R2D:4D and L2D:4D respectively.

Table S24: Robustness Test 5l: Restricted Sample with Mean 2D:4D without Self-employed Farmers (OLS)

	Men	Women	Both
Mean 2D:4D	-0.294	-0.106	-0.196
	(0.229)	(0.188)	(0.146)
Female			-0.023
			(0.014)
Year of Birth	-0.004**	-0.002**	-0.003**
	(0.001)	(0.001)	(0.000)
Below Abitur	-0.004	-0.041	-0.026
	(0.028)	(0.022)	(0.017)
Oth. Higher Educ.	0.025	0.001	0.013
	(0.026)	(0.020)	(0.016)
College	0.077*	0.034	0.055**
	(0.029)	(0.025)	(0.019)
Health	-0.010	0.010	0.002
	(0.013)	(0.010)	(0.008)
Urban	-0.012	0.008	-0.000
	(0.020)	(0.016)	(0.013)
Gross Pers. Inc.	0.008	-0.001	0.007
	(0.006)	(0.009)	(0.005)
Constant	8.786**	4.472**	6.474**
	(1.474)	(1.081)	(0.898)
Observations	1002	1106	2108
Pseudo R^2			
χ^2			
p-value	0.000	0.002	0.000
Adjusted R^2	0.057	0.016	0.041

OLS regressions; robust standard errors in parentheses;
* $p < 0.05$, ** $p < 0.005$.

4 Exploratory analyses

Table S25: Exploratory Analysis: Gender Effect Tests

	Full Sample	Corrected	Restricted
	Coefficient Difference		
R2D:4D	0.156	0.257	0.171
	(0.229)	(0.250)	(0.245)
L2D:4D	0.258	0.305	0.222
	(0.178)	(0.248)	(0.240)
Mean 2D:4D	0.280	0.351	0.287
	(0.251)	(0.298)	(0.301)
	Z-tests		
R2D:4D	0.680	1.027	0.698
L2D:4D	1.453	1.230	0.922
Mean 2D:4D	1.117	1.180	0.951

Notes: Top panel shows 2D:4D coefficient differences between men and women, based on OLS models. Standard errors in parentheses. Bottom panel presents z-scores.

Table S26: Exploratory Analysis: Gender Effect Tests (Without Self-employed Farmers)

	Full Sample	Corrected	Restricted
	Coefficient Difference		
R2D:4D	0.108	0.207	0.122
	(0.226)	(0.248)	(0.243)
L2D:4D	0.215	0.221	0.142
	(0.175)	(0.244)	(0.236)
Mean 2D:4D	0.210	0.251	0.188
	(0.246)	(0.292)	(0.296)
	Z-tests		
R2D:4D	0.475	0.837	0.503
L2D:4D	1.232	0.904	0.601
Mean 2D:4D	0.853	0.860	0.633

Notes: Top panel shows 2D:4D coefficient differences between men and women, based on OLS models. Standard errors in parentheses. Bottom panel presents z-scores.

5 Minimum detectable effect size (power)

Table S27: Minimum Detectable Effect Sizes

Sample:	FS	FS	CS	CS	RS	RS
Confidence Interval:	%0.5	%5	%0.5	%5	%0.5	%5
(M) R2D:4D	0.031	0.024	0.033	0.025	0.033	0.025
(W) R2D:4D	0.026	0.020	0.026	0.020	0.026	0.020
(M) L2D:4D	0.032	0.025	0.033	0.026	0.035	0.027
(F) L2D:4D	0.024	0.019	0.033	0.025	0.031	0.024
(All) R2D:4D	0.020	0.016	0.021	0.016	0.021	0.016
(All) L2D:4D	0.019	0.015	0.024	0.018	0.023	0.018
(M) Mean 2D:4D	0.033	0.025	0.033	0.026	0.034	0.026
(W) Mean 2D:4D	0.025	0.019	0.028	0.022	0.030	0.023
(All) Mean R2D:4D	0.020	0.015	0.022	0.017	0.023	0.017

Notes: FS is the full sample, CS is the corrected sample, RS is the restricted sample; (M) is the abbreviation of men and (W) is of women. Right and left 2D:4D's are shown as R2D:4D and L2D:4D.

6 Not pre-registered robustness tests and exploratory analyses

Table S28: Not Pre-registered Analysis: Only Controlling for Gender

	Both	Both
R2D:4D	-1.362	
	(1.534)	
L2D:4D		-1.393
		(1.166)
Female	-0.470**	-0.446**
	(0.149)	(0.149)
Constant	-0.677	-0.654
	(1.531)	(1.164)
R2D:4D (ME)	-0.116	
	(0.131)	
L2D:4D (ME)		-0.119
		(0.100)
Observations	2151	2156
Pseudo R^2	0.008	0.008
χ^2	11.02	10.87
p-value	0.004	0.004

Logit regressions; standard errors in parentheses;
* $p < 0.05$, ** $p < 0.005$. (ME) shows marginal effects.
Right and left 2D:4D's are represented by R2D:4D and L2D:4D respectively.

Table S29: Not Pre-registered Analysis: Interaction with Age (OLS)

	Men	Women	Men	Women	Both	Both
R2D:4D	0.450	-0.501			-0.055	
	(0.576)	(0.415)			(0.359)	
L2D:4D			0.325	-0.089		0.107
			(0.533)	(0.318)		(0.283)
Female					-0.024	-0.022
					(0.014)	(0.014)
Age	0.017	-0.008	0.016	0.001	0.004	0.007
	(0.013)	(0.009)	(0.011)	(0.006)	(0.008)	(0.006)
R2D:4D × Age	-0.012	0.010			-0.000	
	(0.013)	(0.009)			(0.008)	
L2D:4D × Age			-0.012	0.001		-0.004
			(0.011)	(0.006)		(0.005)
Below Abitur	-0.007	-0.045*	-0.004	-0.046*	-0.028	-0.028
	(0.027)	(0.022)	(0.026)	(0.022)	(0.017)	(0.017)
Oth. Higher Educ.	0.031	0.001	0.030	0.002	0.015	0.016
	(0.026)	(0.020)	(0.026)	(0.020)	(0.016)	(0.016)
College	0.079*	0.035	0.079*	0.039	0.055**	0.057**
	(0.029)	(0.025)	(0.029)	(0.025)	(0.019)	(0.019)
Health	-0.009	0.008	-0.008	0.008	0.002	0.002
	(0.013)	(0.009)	(0.013)	(0.010)	(0.008)	(0.008)
Urban	-0.017	0.008	-0.018	0.010	-0.003	-0.003
	(0.020)	(0.016)	(0.020)	(0.016)	(0.013)	(0.013)
Gross Pers. Inc.	0.010	-0.004	0.010	-0.004	0.007	0.007
	(0.006)	(0.009)	(0.006)	(0.009)	(0.005)	(0.005)
Constant	-0.576	0.451	-0.450	0.037	-0.030	-0.194
	(0.576)	(0.411)	(0.541)	(0.322)	(0.359)	(0.288)
Observations	1021	1130	1027	1129	2151	2156
Adjusted R^2	0.060	0.013	0.061	0.013	0.040	0.040

OLS regressions; robust standard errors in parentheses; * $p < 0.05$, ** $p < 0.005$.
Right and left 2D:4D's are represented by R2D:4D and L2D:4D respectively.

Table S30: Not Pre-registered Analysis: Narrow Definition of Entrepreneurship

	Men	Women	Men	Women	Both	Both
R2D:4D	0.482	-1.360			-0.491	
	(4.759)	(6.885)			(3.876)	
L2D:4D			-0.038	0.191		-1.248
			(4.120)	(3.537)		(2.844)
Female					-0.141	-0.132
					(0.414)	(0.414)
Year of Birth	-0.001	-0.040	-0.000	-0.041	-0.009	-0.009
	(0.018)	(0.032)	(0.018)	(0.032)	(0.015)	(0.015)
Below Abitur	0.284	0.322	0.235	0.320	0.263	0.239
	(1.086)	(1.174)	(1.086)	(1.172)	(0.775)	(0.775)
Oth. Higher Educ.	0.275	-0.374	0.236	-0.305	-0.025	-0.041
	(0.561)	(0.751)	(0.562)	(0.757)	(0.447)	(0.447)
College	-0.628	-3.682*	-0.607	-3.743*	-1.203*	-1.160
	(0.678)	(1.608)	(0.675)	(1.654)	(0.604)	(0.600)
Health	-0.103	0.788*	-0.090	0.817*	0.302	0.317
	(0.291)	(0.319)	(0.291)	(0.324)	(0.211)	(0.211)
Urban	-0.369	-0.673	-0.363	-0.653	-0.437	-0.433
	(0.464)	(0.681)	(0.465)	(0.685)	(0.376)	(0.376)
Gross Pers. Inc.	0.309*	0.941**	0.297*	0.951**	0.475**	0.465**
	(0.118)	(0.210)	(0.117)	(0.214)	(0.097)	(0.095)
Constant	-3.144	72.254	-4.161	71.350	12.886	12.349
	(36.691)	(62.095)	(36.582)	(61.948)	(30.042)	(29.904)
R2D:4D (ME)	0.010	-0.013			-0.008	
	(0.104)	(0.065)			(0.060)	
L2D:4D (ME)			-0.001	0.002		-0.019
			(0.089)	(0.033)		(0.044)
Observations	888	1033	894	1031	1921	1925
Pseudo R^2	0.041	0.265	0.039	0.268	0.094	0.093
χ^2	7.89	32.26	7.53	32.62	29.74	29.44
p-value	0.444	0.000	0.481	0.000	0.000	0.001

Logit regressions; standard errors in parentheses; * $p < 0.05$, ** $p < 0.005$. (ME) shows marginal effects. Right and left 2D:4D's are represented by R2D:4D and L2D:4D respectively.

Table S31: Not Pre-registered Analysis: Probability of Ever Having Been Self-Employed

	Men	Women	Men	Women	Both	Both
R2D:4D	-0.953	0.926			-0.028	
	(1.785)	(1.821)			(1.264)	
L2D:4D			-0.748	0.169		-0.123
			(1.675)	(0.946)		(0.857)
Female					-0.265	-0.266
					(0.139)	(0.139)
Year of Birth	-0.032**	-0.023**	-0.032**	-0.023**	-0.028**	-0.028**
	(0.007)	(0.007)	(0.007)	(0.007)	(0.005)	(0.005)
Below Abitur	-1.511	-1.091*	-1.551	-1.098*	-1.072*	-1.092*
	(1.032)	(0.488)	(1.031)	(0.487)	(0.433)	(0.432)
Oth. Higher Educ.	0.331	-0.168	0.301	-0.197	0.084	0.059
	(0.240)	(0.265)	(0.240)	(0.269)	(0.178)	(0.179)
College	0.750**	0.581*	0.729**	0.615*	0.641**	0.639**
	(0.219)	(0.255)	(0.218)	(0.253)	(0.163)	(0.163)
Health	-0.117	0.121	-0.114	0.111	0.007	0.002
	(0.121)	(0.110)	(0.120)	(0.111)	(0.082)	(0.082)
Urban	-0.178	0.006	-0.170	0.051	-0.094	-0.070
	(0.180)	(0.199)	(0.179)	(0.199)	(0.132)	(0.132)
Gross Pers. Inc.	0.078*	-0.051	0.078*	-0.063	0.061*	0.059
	(0.033)	(0.090)	(0.033)	(0.090)	(0.030)	(0.030)
Constant	61.714**	41.521**	61.143**	42.288**	52.625**	52.584**
	(14.220)	(13.807)	(14.115)	(13.809)	(10.021)	(9.994)
R2D:4D (ME)	-0.119	0.087			-0.003	
	(0.223)	(0.171)			(0.139)	
L2D:4D (ME)			-0.094	0.016		-0.013
			(0.210)	(0.089)		(0.094)
Observations	1021	1130	1027	1129	2151	2156
Pseudo R^2	0.074	0.036	0.072	0.037	0.057	0.056
χ^2	47.88	26.81	47.64	27.93	78.67	78.69
p-value	0.000	0.001	0.000	0.000	0.000	0.000

Logit regressions; standard errors in parentheses; * $p < 0.05$, ** $p < 0.005$. (ME) shows marginal effects. Right and left 2D:4D's are represented by R2D:4D and L2D:4D respectively.

Table S32: Not Pre-registered Analysis: Entrepreneurial Intent (OLS)

	Men	Women	Men	Women	Both	Both
R2D:4D	-18.167	6.658			-5.519	
	(25.552)	(16.091)			(14.134)	
L2D:4D			-21.752	-6.155		-14.251
			(15.575)	(12.589)		(9.979)
Female					-3.361	-3.483*
					(1.731)	(1.724)
Age	-0.145	-0.269*	-0.136	-0.268*	-0.195**	-0.191**
	(0.110)	(0.097)	(0.104)	(0.096)	(0.067)	(0.067)
Below Abitur	1.318	3.661	0.983	3.563	2.854	2.682
	(3.703)	(3.733)	(3.607)	(3.654)	(2.608)	(2.572)
Oth. Higher Educ.	4.786	-0.870	4.140	-0.732	1.691	1.580
	(3.451)	(1.973)	(3.378)	(1.935)	(1.828)	(1.811)
College	10.259**	3.430	9.972**	3.586	6.713**	6.704**
	(3.105)	(2.537)	(3.119)	(2.604)	(2.016)	(2.020)
Health	-0.423	0.733	-0.300	0.697	0.385	0.419
	(1.326)	(1.100)	(1.328)	(1.108)	(0.830)	(0.838)
Urban	-5.614*	-1.832	-6.227*	-1.864	-3.356*	-3.632*
	(2.659)	(2.016)	(2.647)	(2.003)	(1.601)	(1.594)
Gross Pers. Inc.	-0.445	0.231	-0.536	0.220	-0.103	-0.179
	(0.491)	(0.607)	(0.510)	(0.618)	(0.374)	(0.383)
Constant	34.765	7.811	38.672*	20.730	21.199	30.230*
	(27.494)	(18.517)	(16.822)	(17.064)	(15.671)	(12.053)
Observations	243	299	247	300	542	547
Adjusted R^2	0.046	0.021	0.049	0.022	0.041	0.044

OLS regressions; robust standard errors in parentheses; * $p < 0.05$, ** $p < 0.005$.
Right and left 2D:4D's are represented by R2D:4D and L2D:4D respectively.

Table S33: Comparison of Effect Size and Meta Analysis

	R2D:4D		L2D:4D		R2D:4D	L2D:4D
	Men	Women	Men	Women	Both	Both
Standardized marg. effects, our main estim.	-0.0085	0.0011	-0.0156	-0.0003	-0.0033	-0.0060
Standard error	0.0101	0.0077	0.0104	0.0074	0.0063	0.0065
Standard. marg. eff., Nicolaou et al. (2018)	0.0046	-0.0100	-0.0579	-0.0333	-0.0025	-0.0489
Standard error	0.0181	0.0144	0.0206	0.0188	0.0124	0.0153
Difference in the standardized effects	-0.0132	0.0111	0.0423	0.0330	-0.0008	0.0429
Standard error of the difference	0.0208	0.0164	0.0230	0.0201	0.0139	0.0166
z-value for test of difference in the effects	-0.6351	0.6808	1.8357	1.6385	-0.0543	2.5902
MDE (0.5% level) for difference in effects	0.0758	0.0598	0.0841	0.0735	0.0506	0.0605
MDE (5% level) for difference in effects	0.0581	0.0458	0.0645	0.0564	0.0388	0.0464
Combined effect	-0.0054	-0.0014	-0.0242	-0.0047	-0.0031	-0.0125
Standard error of combined effect	0.0088	0.0068	0.0093	0.0069	0.0056	0.0060
Upper limit 99.5% CI of combined effect	0.0194	0.0178	0.0018	0.0145	0.0126	0.0042
Lower limit 99.5% CI of combined effect	-0.0302	-0.0205	-0.0502	-0.0240	-0.0189	-0.0292
Upper limit 95% CI of combined effect	0.0119	0.0120	-0.0060	0.0087	0.0079	-0.0009
Lower limit 95% CI of combined effect	-0.0227	-0.0147	-0.0424	-0.0182	-0.0141	-0.0242

The standardized marginal effects are for a one standardized-deviation change in 2D:4D in the respective samples. The columns titled "Both" provide results from estimations pooling men and women. Nicolaou et al. (2018) did not report these, we estimated the effects based on their data using logistic regressions analogously to those they conducted for men and women separately. MDE stands for minimum detectable effect size and is the difference in effects between the studies that we have 80% power to find in the z-test. The "combined effects" combine the estimated effects of our study and those of Nicolaou et al. (2018) using fixed-effects meta analyses. Right and left 2D:4D's are represented by R2D:4D and L2D:4D, respectively.
